# Identification of Predictive Cis-Regulatory Elements Using a Discriminative Objective Function and a Dynamic Search Space

**DOI:** 10.1371/journal.pone.0140557

**Published:** 2015-10-14

**Authors:** Rahul Karnik, Michael A. Beer

**Affiliations:** 1 Department of Biomedical Engineering, Johns Hopkins University, Baltimore, MD, United States of America; 2 McKusick-Nathans Institute of Genetic Medicine, Johns Hopkins University, Baltimore, MD, United States of America; McGill University, CANADA

## Abstract

The generation of genomic binding or accessibility data from massively parallel sequencing technologies such as ChIP-seq and DNase-seq continues to accelerate. Yet state-of-the-art computational approaches for the identification of DNA binding motifs often yield motifs of weak predictive power. Here we present a novel computational algorithm called MotifSpec, designed to find predictive motifs, in contrast to over-represented sequence elements. The key distinguishing feature of this algorithm is that it uses a dynamic search space and a learned threshold to find discriminative motifs in combination with the modeling of motifs using a full PWM (position weight matrix) rather than *k*-mer words or regular expressions. We demonstrate that our approach finds motifs corresponding to known binding specificities in several mammalian ChIP-seq datasets, and that our PWMs classify the ChIP-seq signals with accuracy comparable to, or marginally better than motifs from the best existing algorithms. In other datasets, our algorithm identifies novel motifs where other methods fail. Finally, we apply this algorithm to detect motifs from expression datasets in *C*. *elegans* using a dynamic expression similarity metric rather than fixed expression clusters, and find novel predictive motifs.

## Introduction

Multiple mechanisms exist to modulate protein levels in a cell and create a dynamic cellular phenotype from a static genotype. One such mechanism is transcriptional regulation. Transcription factors (TFs) bind to intergenic cis-regulatory elements and enhance or inhibit the transcription of their target genes. Identifying the DNA binding specificities of transcription factors is necessary to decipher the regulatory network in the cell, identify disease causing mutations in these elements, and engineer synthetic organisms to perform specific biochemical functions. Several technological platforms can be used to identify the binding specificities of the DNA-binding domains of transcription factors to cis-regulatory elements in DNA. Binding can be directly measured in vivo using chromatin immunoprecipitation followed by microarray hybridization (ChIP-chip) [1] or sequencing (ChIP-seq) [2], or in vitro using protein binding microarrays (PBMs) [3]. Alternatively, gene co-expression implies the binding of TFs to cis-regulatory elements, and thus indirectly indicates binding by a common regulator. The spatial resolution of these technologies are typically unable to resolve short TF binding sites, so motif-finding algorithms are usually used to identify TF binding specificity within the set of longer experimentally determined regions [2–4].

Despite these significant advances in technology, there is still a substantial gap in our ability to generate PWMs that accurately describe binding specificities from these experiments. For instance, 246 candidate DNA-binding proteins from yeast were assayed with PBMs [5]. Of these, predictive motifs were found in only 89 cases, or 36% of the factors assayed. Similarly, 23 transcription factors from *Caenorhabiditis elegans* were assayed using ChIP-seq as part of the modENCODE project [6]. Predictive motifs were found for only 8 (35%) of these factors. Considering the rapid growth of these technologies [6–9], improved algorithms to extract regulatory sequence information from these data sets would be of clear value. In particular, better motif models could improve the identification of regulatory mutations associated with common human disease [10] and could be used to develop improved techniques to detect regulatory variation responsible for differences in gene expression between species [11–13].

Generally, motif-finding algorithms search a set of sequences for shared cis-regulatory elements. We term this set of sequences the search space. Early motif-finding algorithms optimized for over-represented sequence motifs, which are sequence patterns found more often in the search space than would be predicted by a null or background sequence model. Successful algorithms of this class include AlignACE [4] and MEME [14], which use Gibbs sampling and expectation maximization respectively, to search for the optimal sequence motif. A discriminative approach, in contrast, searches for specific motifs, those cis-regulatory elements which are present at a higher frequency in a positive set of sequences than in a negative set of sequences. A frequency difference in the positive and negative sets is required for accurate classification of one set from the other, and is often not reflected by over-representation in the positive set, as many instances of the over-represented motifs are also found in the negative set. Several algorithms of the discriminative type have been developed previously, including Amadeus [15], DREME [16], HOMER [17], Dimont [18], DECOD [19], and others [20–23]. By necessity, a discriminative objective function is more expensive to compute; it requires scoring not only the sequences in the positive search space, but also those sequences in the negative set to establish the scarcity of the motif in negative sequences. Consequently, some previous algorithms have used a *k*-mer sequence model for the motif while performing discriminative motif finding. Our algorithm, which we call MotifSpec, uses a full position-weight matrix as its sequence model, which we show performs better than many existing discriminative models, and is comparable in accuracy to HOMER [17] and Dimont [18].

The positive set can be defined through one of three methods. First, we might have direct binding data for the protein or TF, and the set of sequences whose binding score is above a threshold is used as the positive set. This threshold can be a simple rank, or can result from computation of a p-value given some null binding model. This approach is common with ChIP-chip, ChIP-seq and PBM experiments. Second, we might use co-expression as a proxy for binding, and choose a correlation threshold to define the search space. This method often involves clustering co-expressed genes and then running a motif-finding algorithm on the upstream regions of genes in the individual cluster [4,24–27]. Finally, we might use prior biological annotation to identify bound sequences. In the first two cases, the optimal boundary between the positive and negative sets is generally not obvious, and changing the boundary threshold will change the membership of the positive and negative sets.

Most existing discriminative motif finding algorithms consider the positive and negative search spaces to be fixed. Instead of using a fixed set of positive sequences, a dynamic approach allows the boundary between the positive and negative sequences to evolve during the search procedure. A dynamic threshold can be applied when searching for motifs in any set of continuous enrichment data, such as ChIP-seq peak intensity. MotifSpec uses such a dynamic search space to optimize the specificity of the motif, which we show improves quantitative measures of motif predictive power on PBM and expression datasets.

## Methods

MotifSpec is a heavily modified version of the Gibbs sampling algorithm implemented in AlignACE [26]. The key innovations in MotifSpec are a dynamic search space, a dynamic threshold for sequence score, and a hypergeometric discriminative objective function.

### Dynamic threshold to determine positive and negative sets

As mentioned above, current algorithms typically use a fixed search space. In contrast, MotifSpec dynamically optimizes the threshold defining the positive and negative sets using the objective function discussed below. In the case of TF binding, this threshold is a binding intensity. This dynamic search space is appropriate given that TFs have widely varying target set sizes.

### Dynamic threshold for sequence score

In both AlignACE and MotifSpec, sequence positions are scored according to the equation: *L* = Pr(*S*|*θ*)/Pr(*S*|*θ*_0_), where *L* is the site score or likelihood ratio, *S* is the site being scored, *θ* is a PWM sequence model, and *θ*_*0*_ is a background distribution. This odds-ratio is then converted into a probability using a Bayesian framework. We compute the probability of the PWM model *θ* given the site *S* currently being scored as: Pr(*θ*|*S*) = *L* Pr(*θ*)/(*L* Pr(*θ*) + 1 − Pr(*θ*)).

In AlignACE, a fixed Pr(*θ*|*S*) threshold determines whether a specific site is an instance of the current PWM, and whether it should be added to the PWM for the next iteration. Instead of a fixed threshold, MotifSpec dynamically adjusts this threshold to maximize the objective function discussed below. We call this threshold the "sequence threshold" because it determines how high the sequence of a site must score by the current PWM to be considered an actual binding site, and to be added to the model. In addition, AlignACE has a parameter called *expect*, which is the prior for number of instances of the motif in the search space. This parameter is used to calculate Pr(*θ*) = (*ew* + *x*(1 − *w*)) / *T*, where *e* is expect, *x* is the current number of motif instances, *w* is a weight assigned to the prior and *T* is the total number of positions available in the search space. Instead of relying on the difficult to estimate expect parameter, MotifSpec assumes one motif instance per sequence, replacing *e* with the number of sequences in the current search space, *s*_*1*_.

### Model components and objective function

In order to support the dynamic thresholds for sequence score and search space membership, the MotifSpec motif model consists of a PWM and two additional components:

1. Search space threshold: the minimum binding score that a sequence must have to be included in the search space or positive set. The set of sequences that are above this threshold is the set *s*_*1*_. In the case of ChIP-seq, this threshold score is read depth; for PBM, it is the binding intensity of the oligo; in the case of expression similarity, it is a correlation measure.
2. Sequence score threshold: the minimum site score that a site in a sequence must achieve to be considered an instance of the motif. The set of sequences that have a site scoring above this threshold is the set *s*_*2*_.

MotifSpec uses a discriminative objective function called a specificity score, which measures the enrichment for sequences to be in both *s*_*1*_ and *s*_*2*_. Given *x* sequences that are in the intersection of the above sets, and *N* total sequences in the positive and negative sets, the specificity score is defined using the hypergeometric distribution as

$$\text{Specificity score}=-\log\left(\sum_{i=x}^{\min(s_{1},s_{2})}\frac{C(s_{1},i)C(N-s_{1},s_{2}-i)}{C(N,s_{2})}\right)$$

This specificity score is the negative logarithm of the group specificity score used by Hughes et al [4]. Since N is typically large for genomic datasets, we calculate the summation in the specificity score in log space using log(*x* + *y*) = log(*x*) + log(1 + exp(log(*y*) − log(*x*))), where *x* and *y* are individual probability terms for different values of *i* in the equation above.

### Weighted PWM

AlignACE and most other motif finders use an equal contribution from each motif instance to compute the PWM. MotifSpec weights the contribution of each instance to the PWM according to the binding score of that instance. Say that we have *n* instances of a motif. Let **w** be the vector of binding scores of the sequences normalized to be between 0 (low) and 1 (high). Assume that the PWM has *k* columns and that *Ii*,*j*,*b* is the indicator variable of having the base *b* at position *j* in instance *i* of the motif. Then the probability *fj*,*b* of having base *b* at position *j* in the motif in an unweighted PWM is $f_{j,b}=\left(\sum_{i=1}^{n}I_{i,j,b}\right)/n$. Instead, MotifSpec calculates the weighted PWM using $f_{j,b}=\left(\sum_{i=1}^{n}I_{i,j,b}w_{i}\right)/\left(\sum_{i=1}^{n}w_{i}\right)$.

### Algorithm

**Fig 1:**
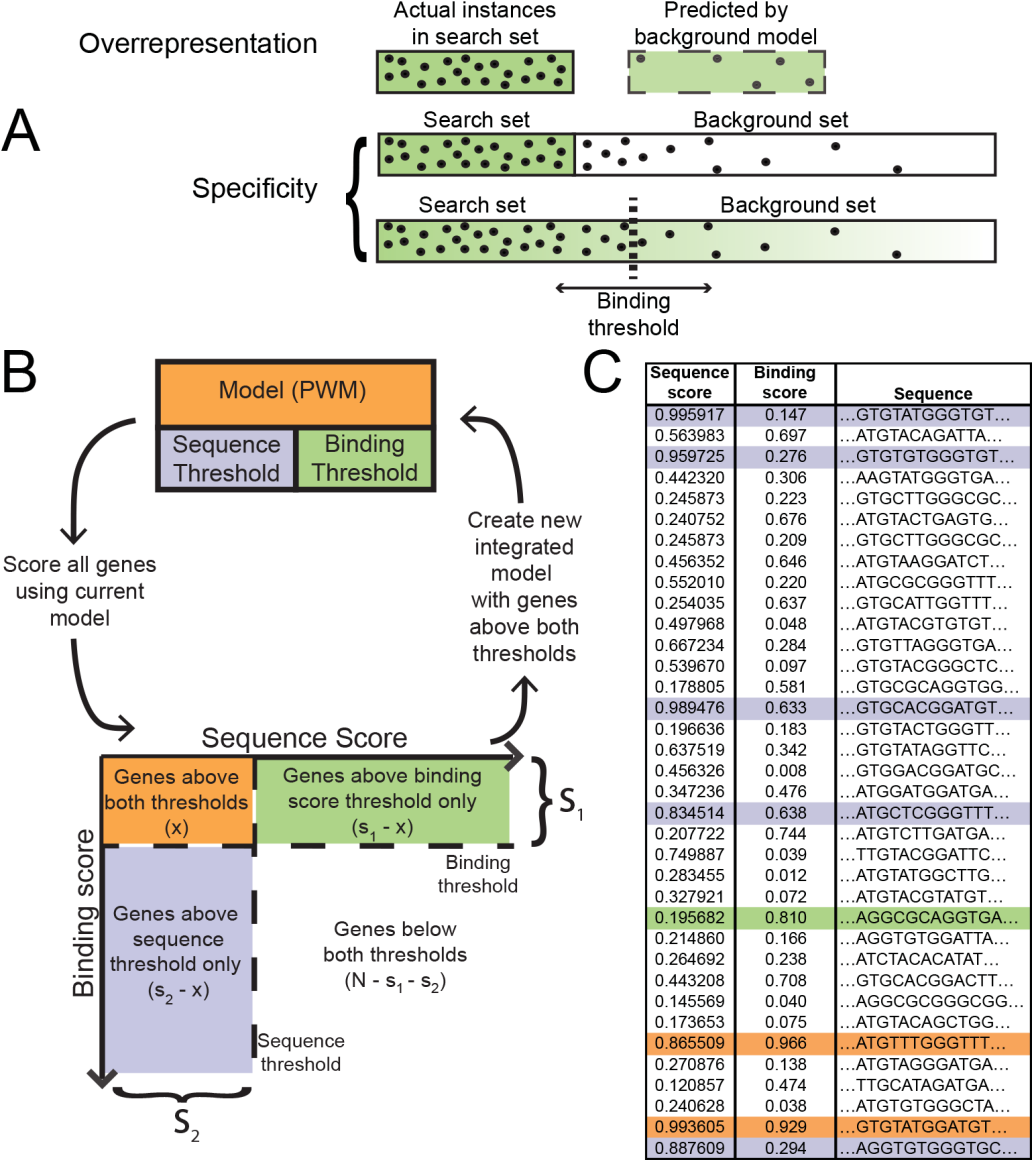
MotifSpec optimizes for specificity rather than over-representation and uses a dynamic search space. (A) An over-represented motif is found in the search space more often than expected according to some background model. It is not necessarily predictive. A specific motif is found in a much higher frequency in the search space than in the background sequences. A dynamic search space threshold finds the optimal search space such that the motif is most discriminative. (B) A schematic of the MotifSpec algorithm. The PWM model is initialized with a random sequence and position in the search space. The model is iteratively refined and the motif and binding score thresholds are adjusted at convergence to maximize specificity. (C) An example of sequences scored using the model. Each sequence has a motif score and a binding score. The binding score determines if a sequence is in the search space. The motif score determines if the sequence has an instance of the motif. The sequences are color-coded according to the set to which they belong as defined in (B).

The MotifSpec algorithm iteratively optimizes the PWM and the thresholds in the model (Fig 1). MotifSpec initializes the model by choosing a random site from the positive search space. Similar to AlignACE, convergence is measured by improvement in the specificity score; MotifSpec stops iterating after a series of iterations without improvement have occurred, with the number of iterations being a customizable parameter. MotifSpec then alternately adjusts the binding score threshold and sequence score threshold to maximize the objective function, given the current PWM. Sites are rescored using these new thresholds. With the new thresholds, the iteration process is repeated, again until *minpass* iterations without improvement occur, and the current model is output. After the first motif is found, subsequent searches are performed with new random starts, terminating early if the current model is similar (CompareACE score greater than a threshold, default 0.9) to a motif previously found with a higher specificity score. The number of such restarts is *s*_*1*_ */(w k)*, where *s1* is the size of the search space (in base pairs), *w* is the number of columns in the PWM motif model, and *k* is a sampling parameter.

### Other Algorithms

All algorithms were run in discriminative mode and with default parameters unless otherwise noted here. HOMER version 4.7 was downloaded from http://homer.salk.edu/homer/download.html and run with motif lengths 8,10,12,14,16,18, and 20, and the highest scoring motif is used for comparison. Dimont was downloaded from http://www.jstacs.de/index.php/Dimont/Download, and was run in discriminative mode by giving each positive sequence a “signal” of 1000, each negative sequence a “signal” of 0, and used a “peak” in the center of each sequence. DECOD version 1.01 was downloaded from http://sb.cs.cmu.edu/DECOD/, and was run with motif width 10 and 12, as larger width required excessive memory (>100GB). Amadeus was downloaded from http://acgt.cs.tau.ac.il/amadeus/. DREME and MEME were run from the MEME suite version 4.10.1 downloaded from http://meme-suite.org/. Weeder was downloaded from the ModTools site (http://159.149.160.51/modtools/). Seed-and-Wobble motifs were downloaded from Uniprobe [9]. Only MEME, Weeder, and AlignACE were run in non-discriminative mode.

### Human, Mouse, and Worm ChIP-seq datasets

We analyzed 16 mammalian ChIP-seq datasets. Three of the datasets (CTCF [27], NRSF [2], and the estrogen receptor (ER) [28]) measured binding in human cells, while the other 13 measured binding of mouse TFs in embryonic stem cells [29]. For the human TFs, we downloaded the raw data from the Gene Expression Omnibus (GEO) database. We re-processed the raw data using the MACS algorithm [30] with default parameters and designated high confidence peaks (false discovery rate < 0.01%) as the positive set. This process resulted in positive sets with 5444, 2417 and 1225 peaks for CTCF, NRSF and ER respectively. A 300 bp window of genomic sequence around each peak was used for analysis for ER and CTCF, a 500bp was used for NRSF. For mouse embryonic stem cell datasets, we used the set of bound sequences defined in [29] as the positive set. We also analyzed ChIP-seq data for *C*. *elegans* from the modENCODE project [6]. We used the peaks designated “appropriate for downstream analysis" in [31] as positive sets. For each positive set, we created a negative set using a previously published procedure that matched the sequence lengths, GC content and repeat fraction of the positive set [32]. We have the freedom to select larger negative sets which usually lead to more predictive motifs. For the human and mouse datasets we used 2x each positive set and for worm used 4x each positive set.

### Yeast PBM datasets

We downloaded data from 132 PBM experiments with 89 yeast TFs from Uniprobe [9]. The raw scores for the approximately 40,000 60-mer probes were translated to strictly positive values and fitted with a log-normal null distribution. The goodness of fit of the scores to the model was verified with qq-plots (S1 Fig). We used 1-*F* as a normalized (0; 1) binding score for each probe, where *F* is the cumulative distribution function of the log-normal translated scores. When learning motifs with MotifSpec, we dynamically optimized the positive and negative sets. For subsequent ROC curve analysis to compare the predictive power of MotifSpec motifs to previous approaches, we used normalized binding scores greater than 0.9 to define the positive set, and binding scores less than 0.5 for the negative set. Our comparative analysis is insensitive to these cutoffs.

### Synthetic sequence-expression data

To test our dynamic expression clustering, we constructed a synthetic dataset consisting of 5000 sequences with lengths sampled from a Gaussian distribution with mean 800bp and standard deviation 100bp, and which have the same GC content as yeast intergenic sequences. Instances of test motifs were seeded into these sequences. To approximately reproduce the structure of yeast intergenic regions, we seeded two classes of motifs: "functional" and "non-functional". Four "non-functional" motifs were seeded into each sequence at random, but the "functional" motifs were only seeded into co-expressed sets of genes. These sets were of varying size, and one "functional" motif was seeded into each member of a co-expressed gene set. A total of 80 functional motifs were seeded. To mimic the dominant yeast expression patterns of stress-induction and stress-repression, 40 of the functional motifs were seeded into stress-induced gene sets, and 40 were seeded into stress-repressed gene sets. Individual expression patterns were sampled from a Gaussian distribution with means centered on these two anti-correlated expression patterns. This procedure produced pairwise correlations between expression pattern means that matched the actual expression data described below.

### Yeast and *C*. *elegans* sequence-expression datasets

We extracted upstream sequences for all yeast (*Saccharomyces cereviseae*) ORFs as previously described [25]. We also created a combined gene expression dataset from three different yeast studies, which included cell cycle timepoints and various metabolic stimuli, for a total of 5228 genes across 292 conditions [33–35]. Similarly, we extracted upstream sequences for all genes in *C*. *elegans* as previously described [25] and combined expression data from three different studies in *C*. *elegans* [36–38] for a total of 82 conditions and 5691 genes.

### Evaluation of motifs for ChIP-seq and PBM datasets

To measure the predictive power of our motifs and compare to those previously reported, we scored the positive and negative sets with each PWM using ScanACE, and ranked all sequences according to the highest scoring site in each sequence. Using this ranked list, we plotted a receiver operator characteristic (ROC) curve and used the area under the ROC curve (auROC) as a measure of how well a motif was able to discriminate between the positive and negative sets.

### Background model

AlignACE uses a single nucleotide frequency model (equivalent to a 0th order Markov model) to calculate the site score. Later algorithms (e.g. [14]) have shown that the use of a higher order background model can prove beneficial. In keeping with this trend, MotifSpec can use a background Markov model with order up to 5. In practice, we tend to use a 3rd order Markov model. Increasing the order of the background model did not result in consistent improvement in performance (S2 Fig). Since our objective function penalizes motifs according to their actual frequency in the negative/background set, it is likely that the background model, which is a summary statistic of the background set, is not as important to performance.

### Gapped motifs and number of PWM columns

One advantage that AlignACE provides over competing algorithms is the ability to find gapped motifs. The PWM model is not necessarily contiguous bases and can actually include gaps. For example, the width of the motif might be 15 bases, but only 10 of these bases might be informative and therefore included in the PWM. This gapped PWM model is useful for TFs that bind as a dimer to two sets of constrained DNA bases separated by unconstrained bases. For example, the yeast TF Gal4 binds to the sequence pattern CGGnnnnnnnnnnnGGC. MotifSpec takes the gapped motif concept one step further: it has the ability to add or remove columns to the PWM randomly during the motif search to see if it improves the specificity score. We use this setting for genome-wide searches using expression data, since we are looking for novel motifs that may have an unpredictable number of informative columns.

### Performance heuristics

Given the definition of the specificity score, it is obvious that each iteration of the MotifSpec algorithm requires the scanning of *N* total sequences for motif instances, unlike over-representation based algorithms such as AlignACE and MEME which would scan the search space alone. Since each random start of the MotifSpec algorithm is independent, however, we can parallelize the searches and essentially reduce running time by a factor equaling the number of parallel instances of MotifSpec (S3 Fig).

Another heuristic used to increase scanning performance is the maintenance of a list of the highest scoring site in each of the input sequences. Since only the highest scoring site determines membership in the set of sequences containing an instance of the motif, we do not need to scan every position of every sequence. Instead, we only scan the position that was the highest scoring site in each sequence in the previous iteration. If the PWM has changed considerably from the previous iteration, MotifSpec then triggers a full scan of all positions in all sequences.

### Software availability

Source code for MotifSpec and documentation describing installation and operating instructions are available from our website: http://www.beerlab.org/motifspec and at https://github.com/rakarnik/motifspec.

## Results

### Human ChIP-seq datasets

**Fig 2:**
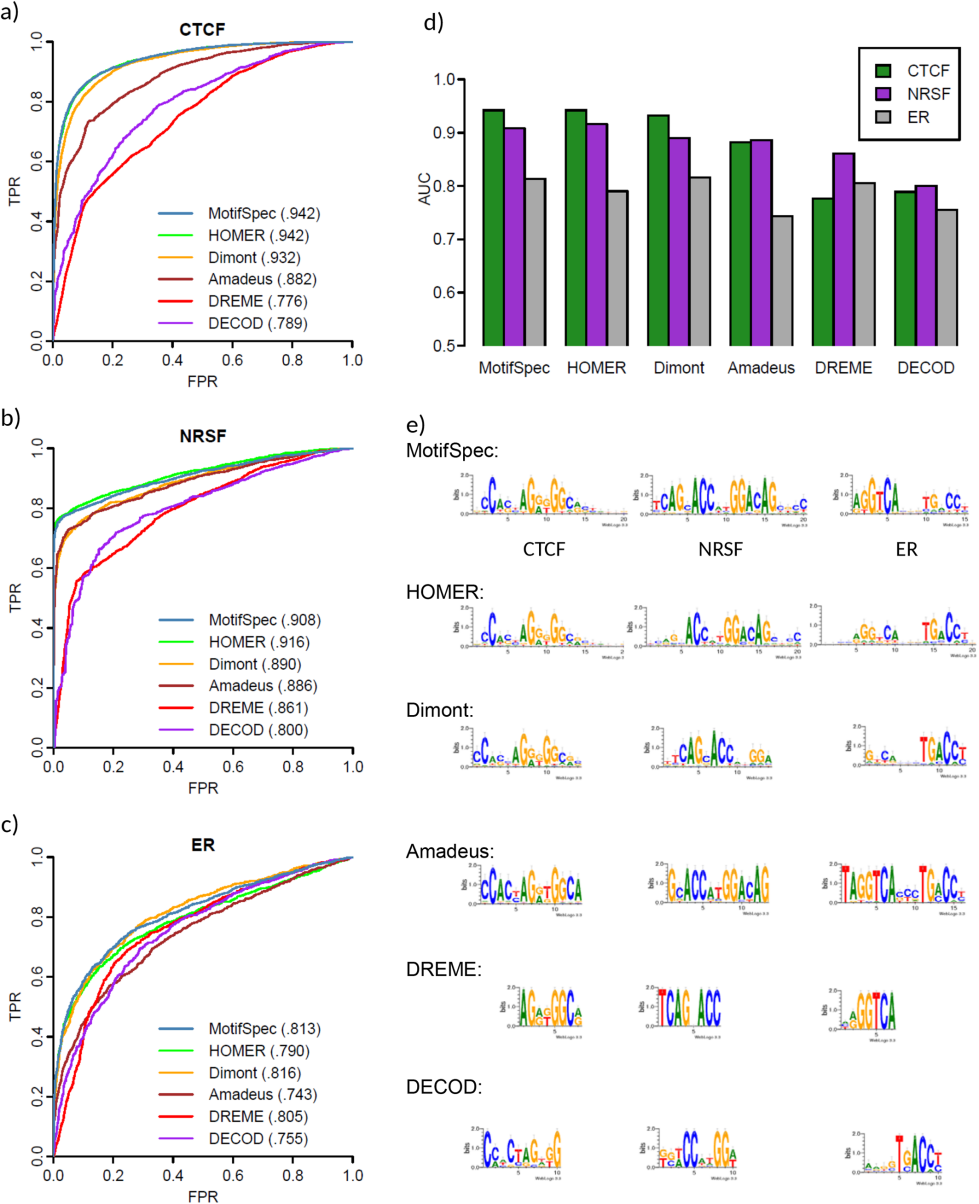
Human ChIP-seq results. MotifSpec performs comparably to HOMER and Dimont and consistently better than DECOD, DREME, and Amadeus in finding a discriminative motif when run on ChIP-seq data for three human transcription factors, CTCF, NRSF and the estrogen receptor (ER). Panels a, b, and c show the ROC curves and auROC values for the top scoring motif from each program when run on the three datasets. Panel d shows a summary comparison of auROC for each algorithm and motif, and panel e shows the top scoring motif found by each program.

We first used ChIP-seq data for three human transcription factors: CTCF, NRSF, and the estrogen receptor (ER), to compare the performance of MotifSpec to five other algorithms: DREME [16], Amadeus [15], HOMER[17], Dimont[18], and DECOD[19]. Positive and negative sets were identical for each method and chosen as described in Methods. In all three cases, the top motif found by MotifSpec was able to discriminate significantly better between the positive and negative sets (higher auROC) than DREME and Amadeus and DECODE, and was generally similar or marginally better than HOMER and Dimont (Fig 2, S4–S6 Figs) The motifs found by each algorithm are shown in Fig 2E. MotifSpec consistently found longer motifs than either DREME or Amadeus. As these algorithms are word-based, we speculate that finding high-scoring exact matches to individual long *k*-mers or regular expressions is less likely and that these long words get filtered out at an early stage in the algorithm. In contrast, as an entirely PWM-based algorithm, MotifSpec is able to detect these longer motifs with gaps and degenerate positions. PWM models typically have more parameters than word based models. To ensure that the better discriminative power of the motifs found by MotifSpec was not due to over-fitting, we performed 5-fold cross-validation on the three datasets using MotifSpec. The motifs found were almost identical to those found using each complete dataset, and the auROC for the test sets were consistently within 1% of the auROC found on the whole dataset.

### Mouse ChIP-seq datasets

**Fig 3:**
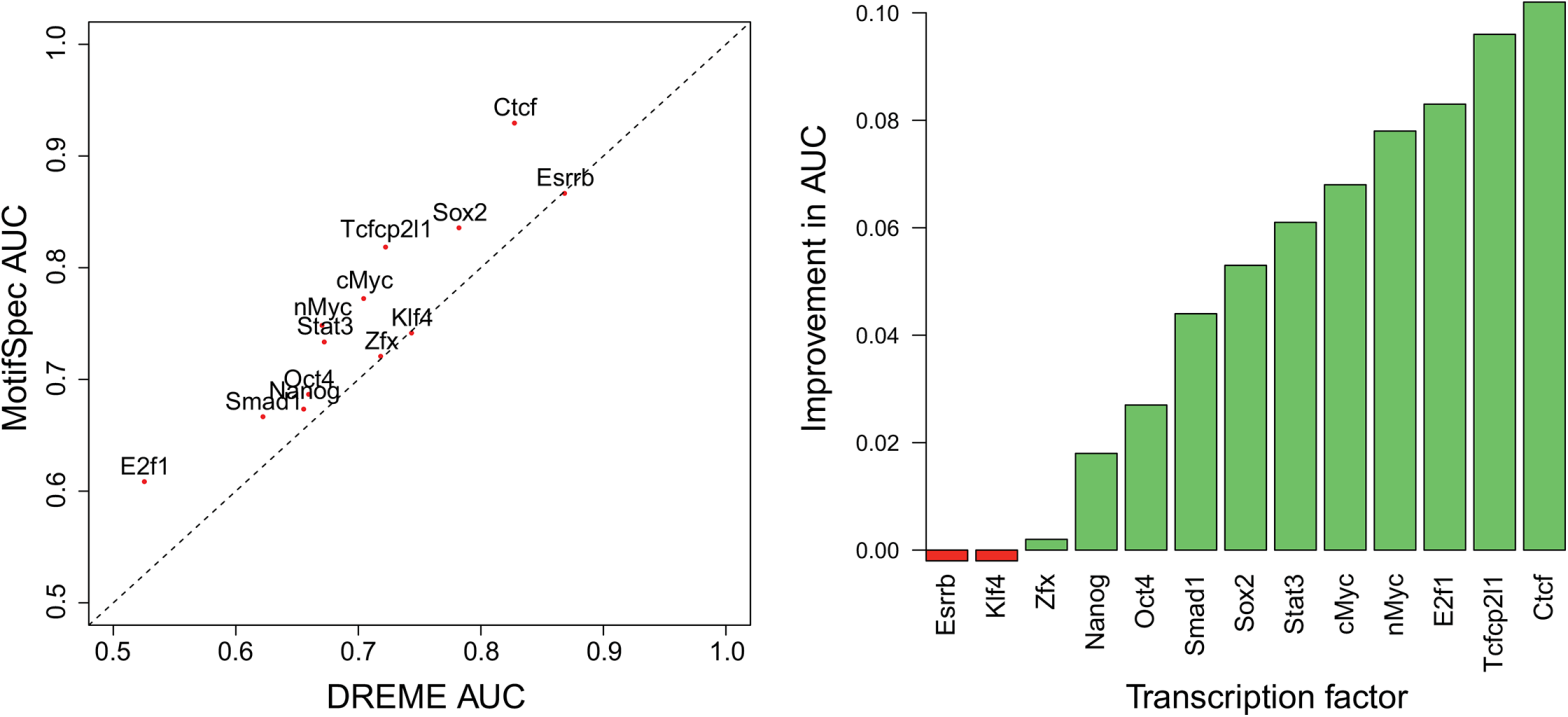
Mouse ChIP-seq results MotifSpec outperforms DREME when run on ChIP-seq data for 13 transcription factors from mouse embryonic stem cells. The left panel shows a plot of the AUC for the top motif reported by MotifSpec against the AUC for the top motif reported by DREME, while the right panel shows the improvement in AUC for the MotifSpec motif relative to the DREME motif.

We next ran MotifSpec on 13 TF ChIP-seq data sets generated in mouse embryonic stem cells [29], and compared our motifs to the previously published motifs found using DREME [16]. Motifs found by MotifSpec were similar, but not identical, to those found by DREME in each dataset. We evaluated the ability of the top motif in each dataset to recover positive sequences from each dataset as above using auROC. In 10 of the 13 datasets, the top motif found by MotifSpec had higher auROC than the top motif found by DREME (Fig 3). In the three remaining cases (Zfx, Klf4, Esrrb), the auROCs were almost identical. For some cases, the auROC values for the best single motifs are low, indicating more combinatorial regulation by those factors.

### *C*. *elegans* ChIP-seq datasets

**Fig 4:**
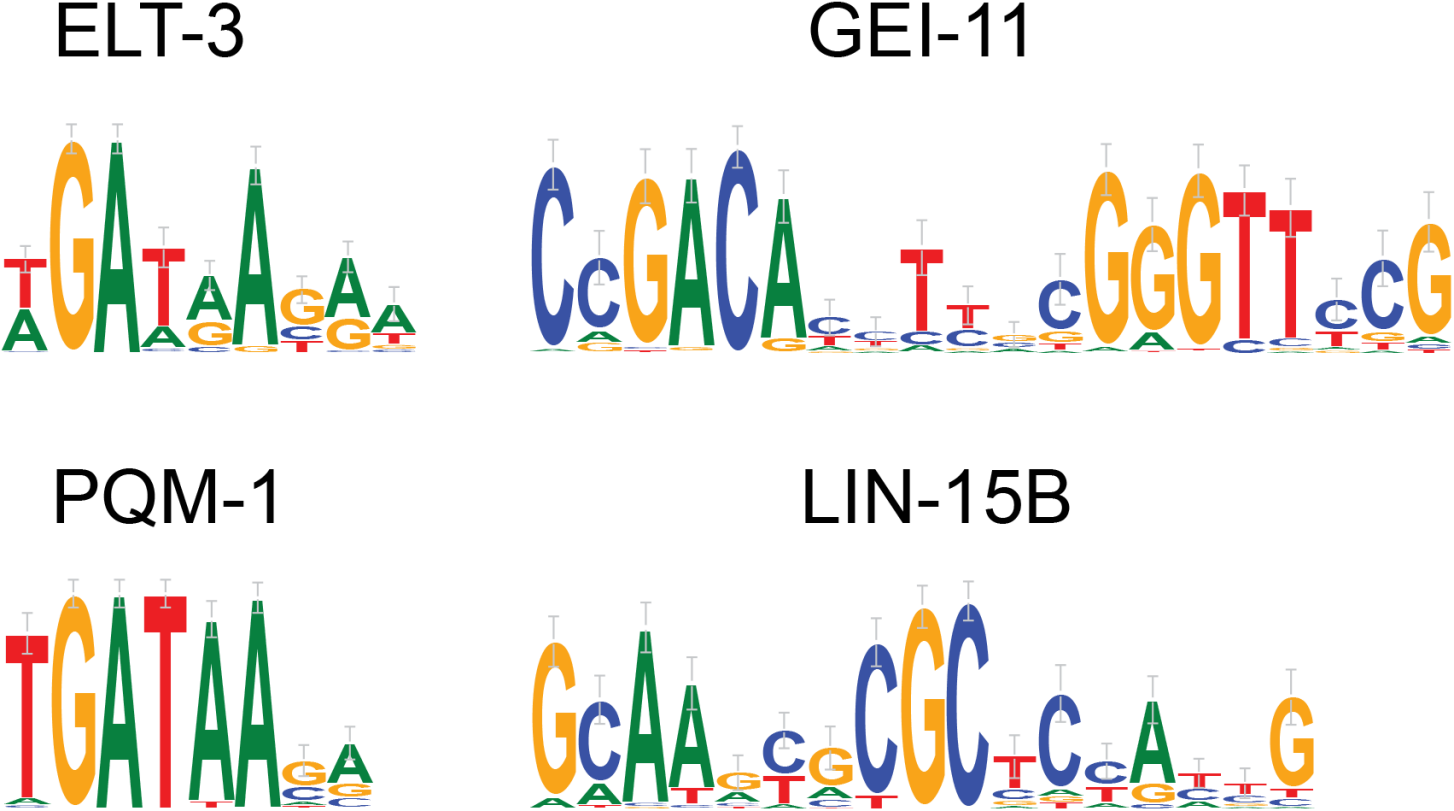
modENCODE ChIP-seq results. Binding specificities for four *C*. *elegans* transcription factors as learnt from ChIP-seq data from the modENCODE project.

We ran MotifSpec on the ChIP-seq data for 23 worm transcription factors [31]. We found four additional motifs, for ELT-3, GEI-11, LIN-15B, and PQM-1, which were not reported in the original analysis (Fig 4). The motif found for LIN-15B was also one of the top motifs found by dynamic expression clustering as described below.

### Yeast PBM datasets

**Fig 5:**
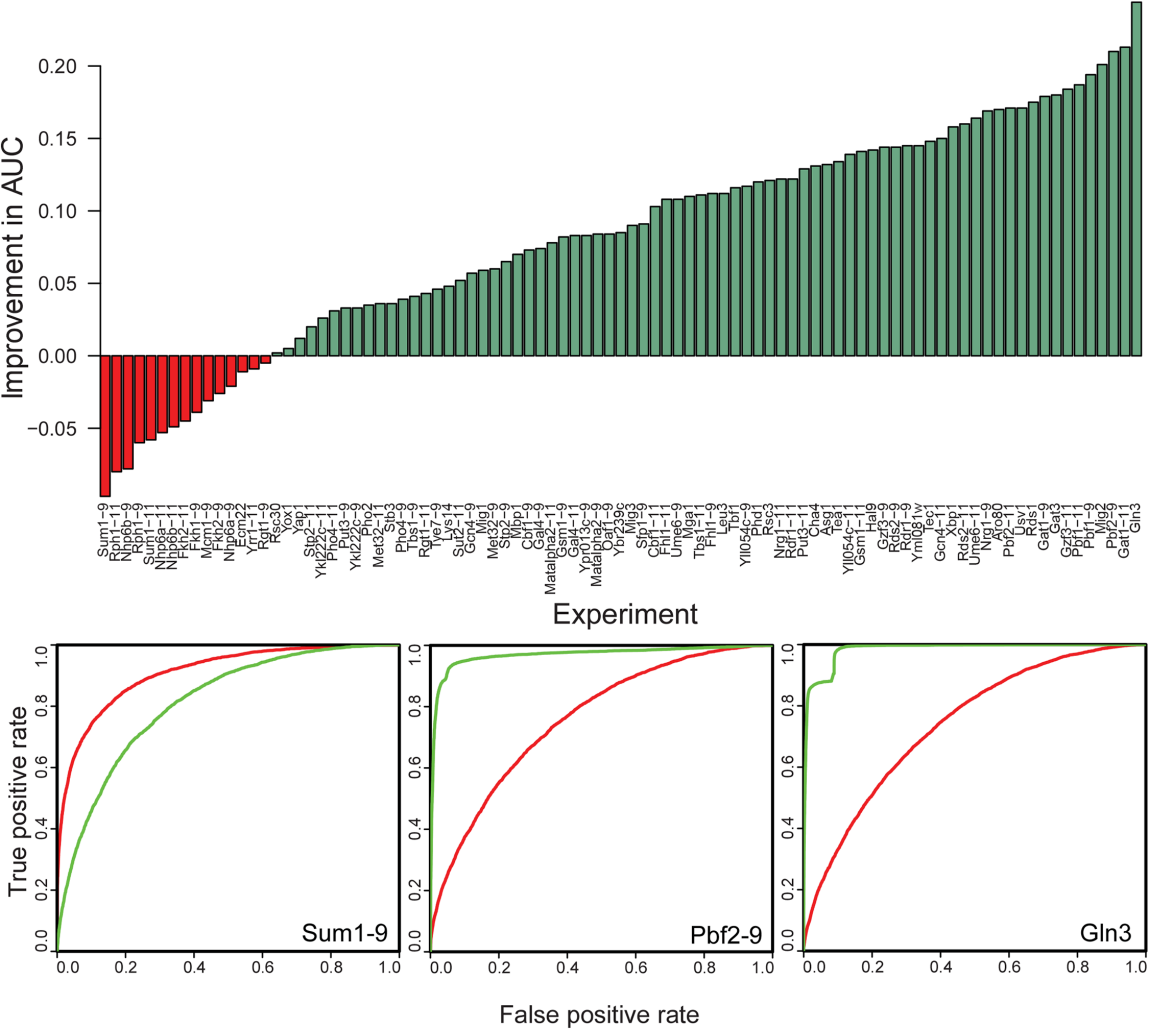
Motifs found by MotifSpec perform better at retrieval of bound probes than the motifs found by Seed-and-Wobble. The barchart shows the percentage improvement in the area under the receiver-operator characteristic (ROC) curve, and the top motif found by MotifSpec performs better than the Seed-and-Wobble motif in the majority of cases where either motif has an AUC of 0.75 or better. Three representative ROC curves are shown, two (Gln3 and Pbf2-9) in which MotifSpec outperforms Seed-and-Wobble and one in which Seed-and Wobble is better (Sum1-9). The red curve is the ROC for the Seed-and-Wobble motif and the green curve is the ROC for the best MotifSpec motif.

We next used MotifSpec to find motifs in the yeast PBM data [5]. We ran MotifSpec using the dynamic search space mode, using normalized binding scores as described in Methods. Using the list of probes ranked by sequence score, we plotted the ROC curve for each TF-microarray pair and calculated auROC. Using this benchmark, we compared the motifs found by MotifSpec to the motifs reported in the original study as found by the Seed and Wobble algorithm [3,5]. We excluded 41 of the 132 experiments where either auROC was less than 0.75, thereby eliminating any experiments where neither algorithm found a sufficiently predictive motif. As shown in Fig 5, the motifs found by MotifSpec outperformed those found by Seed-and-Wobble in 76 of the 91 experiments (83%). This performance improvement was consistent, regardless of the p-value threshold used to define the positive set. With p-value thresholds of 0.05 and 0.01, motifs found by MotifSpec were more predictive in 83/104 (80%) and 93/119 (79%) experiments respectively (S7 Fig).

### Dynamic expression clustering

Previous studies have attempted to detect regulatory elements by identifying sets of co-expressed genes and searching for shared sequence motifs in the upstream regions of these sets of genes [4,25,26]. These efforts first clustered genes by their expression, then used motif-finding algorithms on the upstream regions of each cluster. Given inherent biological and experimental noise, these methods are limited in their ability to tease apart similarly expressed regulatory programs, as similarly expressed genes can be assigned incorrectly to clusters and lower the sensitivity of the subsequent motif-finding step. MotifSpec can increase sensitivity relative to this two-step approach by using its dynamic search spaces to search for elements in co-expressed gene sets without starting with predefined co-regulatory clusters. MotifSpec identifies genes that have similar expression profiles and a shared sequence motif in their upstream regions, and iteratively refines the model of both the expression profile and the sequence motif. This approach was used to search for regulatory elements in genome-wide datasets using combined sequence and expression data. We first test MotifSpec on simulated expression and sequence data, and then on actual yeast expression and sequence data, as it has been emphasized that it is significantly more difficult to detect motifs in actual genomic sequence [39].

### Synthetic sequence-expression dataset

**Fig 6:**
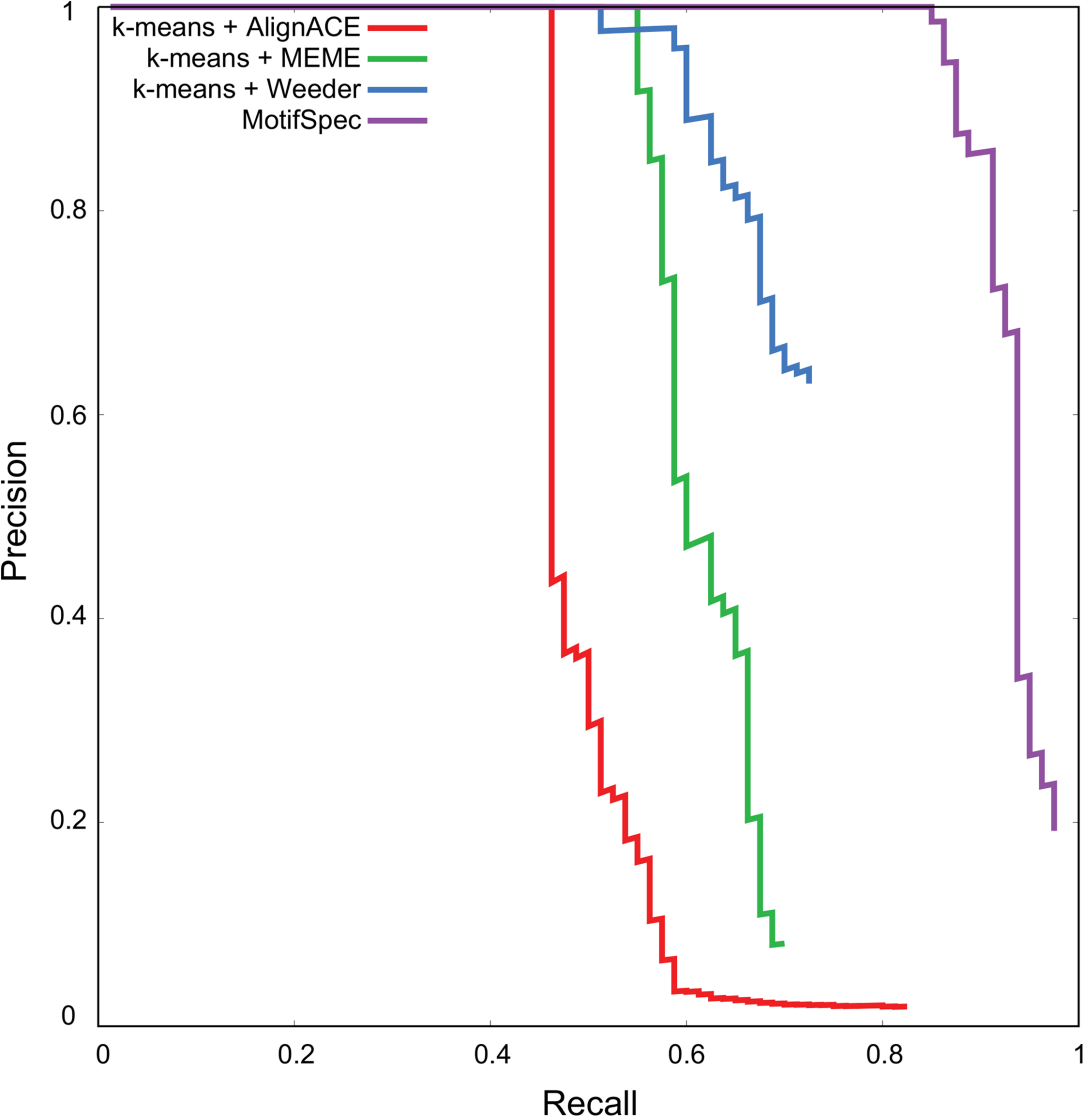
MotifSpec performs better at recovery of seeded motifs from a synthetic sequence-expression dataset than two-step procedures of k-means clustering and motif-finding using AlignACE, MEME and Weeder.

To evaluate the performance of this approach on a simulated dataset, we ran MotifSpec against the synthetic data described in Methods. To compare MotifSpec to the two-step approach, we also clustered the genes using k-means clustering on the expression data and ran the motif finding algorithms AlignACE, MEME and Weeder on the upstream sequences of the genes in each cluster. We evaluated the ability of these distinct methods to recover seeded motifs from the upstream sequences. MotifSpec performed much better than the two-step approach, as shown by the precision-recall curve (Fig 6). At a 10% false positive rate (90% precision), MotifSpec recovered 85% of the seeded motifs, while the two-step algorithms recovered between 46% and 60%.

### Yeast sequence-expression data

**Fig 7:**
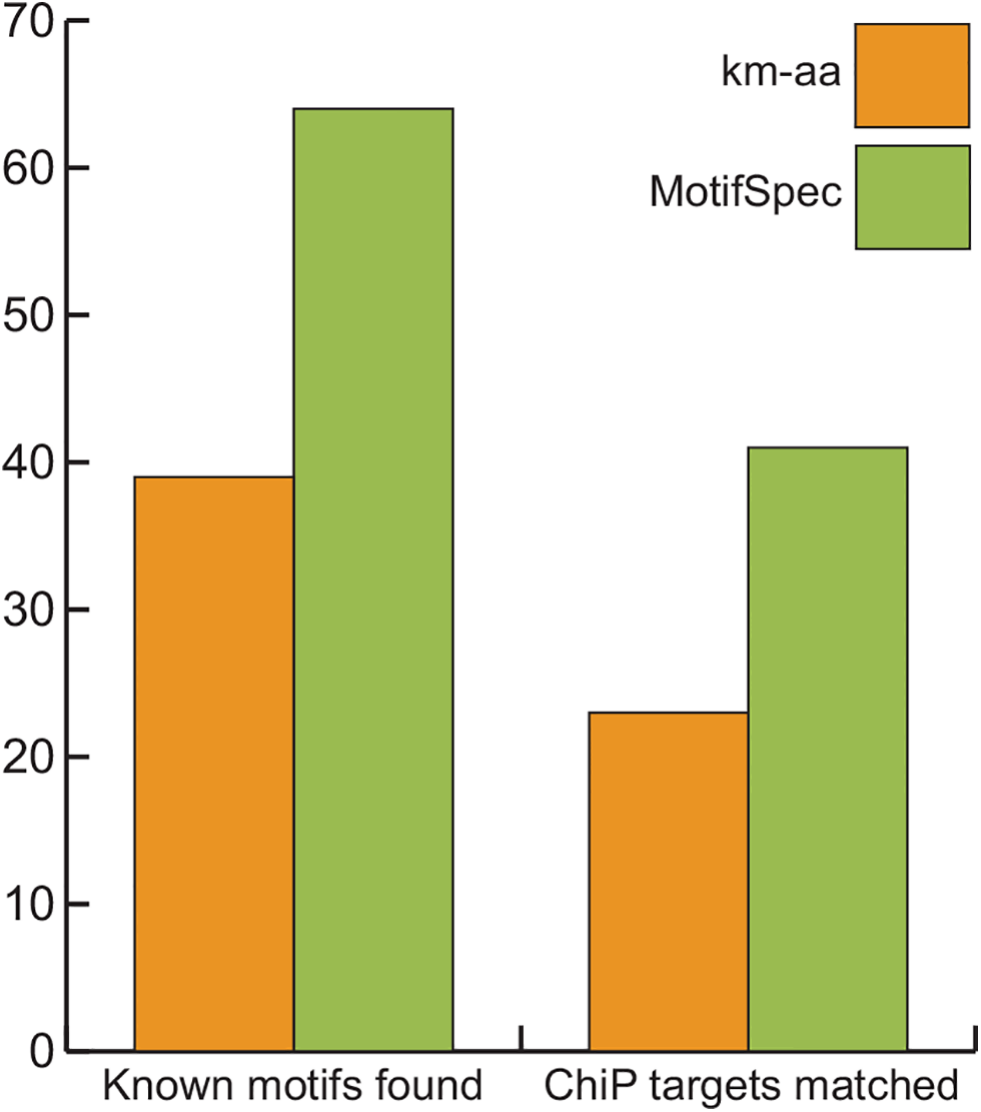
MotifSpec detects more known yeast motifs than the combination of k-means clustering and AlignACE (km-aa). There were 97 known motifs in total. A CompareACE motif similarity score of 0.75 or greater was considered a match. ChIP target sets were considered a match if the hypergeometric p-value for overlap was less than 10^−7^.

We next compared the performance of MotifSpec to that of a two-step process of clustering using k-means and motif-finding within clusters using AlignACE on the actual yeast expression data described in Methods. The list of motifs generated by each algorithm was compared to a compendium of 97 known yeast motifs. MotifSpec found more known motifs from the expression data (65/97 or 67%) than k-means-AlignACE (39/97 or 40%) at a CompareACE threshold of 0.75 (Fig 7). We also compared the target list for predicted motifs with lists of target genes from yeast ChIP-chip data. More target lists found by MotifSpec overlapped significantly (p-value < 10^−7^) with ChIP-chip target lists than those found by k-means-AlignACE (Fig 7).

### *C*. *elegans* sequence-expression data

We next used dynamic expression clustering to find motifs using MotifSpec on *C*. *elegans* expression data. To determine a significance threshold for reporting motifs, we repeated the search on randomized sequences as a negative control. At a specificity score of 26 or higher, we found 135 motifs found in the real dataset and only 10 motifs in the randomized dataset, which translates to a false discovery rate of 7.4%. To generate a non-redundant list, we removed motifs that had a target gene overlap of 30% or greater with a similar motif, leaving 87 motifs total (S1 Table). For each of these motifs, we calculated Gene Ontology (GO) and Anatomy Ontology (AO) [40] enrichment.

**Fig 8:**
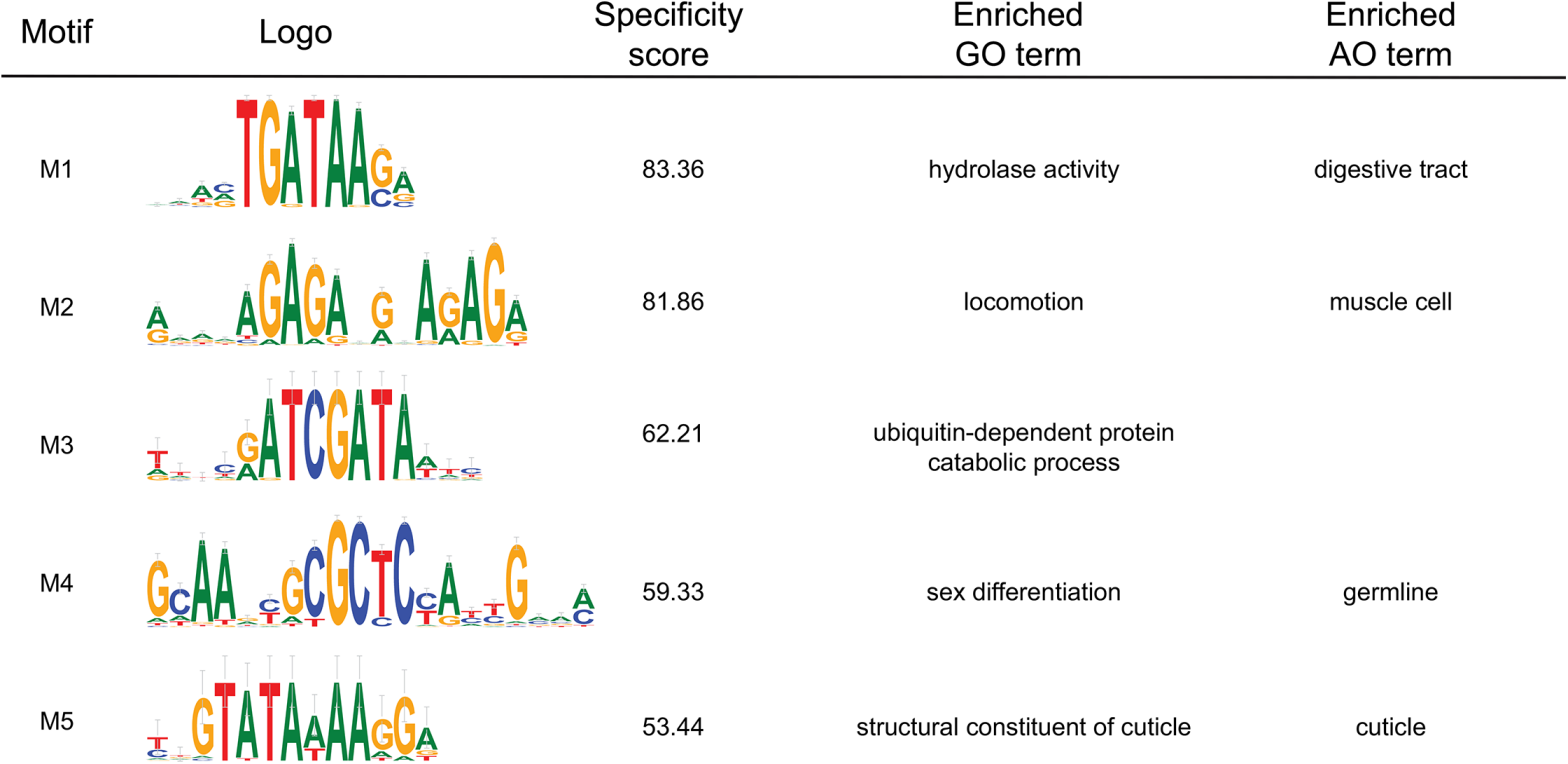
The top 5 motifs found by MotifSpec in a genome-wide search of a *C*. *elegans* sequence and expression dataset. Alongside each motif is its specificity score and any Gene Ontology (GO) and Anatomy Ontology (AO) terms that were enriched in the list of target genes.

The top 5 motifs from this analysis are shown in Fig 8, along with any enriched GO and AO terms. Motif M1 is the known GATA factor binding site. As expected from its intestinal function, GO terms such as "small molecule metabolic process" and "hydrolase activity" are highly enriched in the set of target genes, while the AO terms "digestive tract" and "intestine" are also enriched. Motif M2 was previously found to be associated with the expression of muscle genes [41], and "locomotion" and "muscle cell" are the most enriched ontology terms. We note that several similar GA-rich motifs on our full list (S1 Table) also have high specificity scores and highly overlapping target gene sets, suggesting that the motif may be more degenerate than the highest scoring motif would suggest. Motif M3 is novel and is significantly enriched for "ubiquitin-mediated proteolysis". Its target genes include several F-box family proteins and non-coding RNAs. Motif M4 matches a motif identified as the binding site for CEH-30 from modENCODE ChIP-seq data [6], while our own analysis of the LIN-15B ChIP-seq dataset from the modENCODE project identifies it as well (Fig 4). CEH-30 ensures survival of male-specific neurons during development [42] and M4 targets are enriched for "sex differentiation", which would support the hypothesis that CEH-30 binds to this motif. LIN-15B is implicated in the development of vulval cells [43]. In either case, it is likely to be functional. Motif M5 is another novel motif and targets "cuticle" genes, including 12 collagen and 3 vitellogenin genes.

## Discussion

We have described a novel discriminative motif finding algorithm which uses dynamic search spaces and we evaluated the discovered motifs' predictive performance using ROC analysis. Our algorithm, MotifSpec, showed comparable or marginally improved performance compared to HOMER and Dimont, and markedly improved performance relative to other discriminative motif finding algorithms such as DECOD, DREME and Amadeus when using a fixed search space on mouse and human ChIP-seq data. Since these discriminative motif finders all use similar objective functions, we attribute most of the improvement to our use of a PWM motif model rather than *k*-mers or regular expressions. We also analyzed PBM binding data, and here MotifSpec consistently outperformed the platform-specific motif finder Seed-and-Wobble. Seed-and-Wobble is geared specifically towards the analysis of PBM datasets and uses a *k*-mer enrichment score. MotifSpec is able to do better than Seed-and-Wobble, while remaining agnostic to the underlying technology.

We also presented MotifSpec's novel dynamic expression clustering mode, where we used MotifSpec to search for proximal cis-regulatory elements in yeast and *C*. *elegans* using sequence and expression similarity on an equal footing. Using this approach on worm, we found a non-redundant list of 87 motifs, which are highly specific for gene target co-expression and functional enrichment. These putative cis-regulatory elements are prime candidates for experimental verification. Only 49 of these elements were found in our earlier investigation of worm expression data [25], and only 12 are in the list of 61 motifs found by the FIRE algorithm [44]. Two elements that we have experimentally validated (data not shown) have been shown to be functional in the worm germline [45].

Recently, significant progress has been made detecting TF-binding sequence signals in more complex mammalian enhancers using SVMs [32,46–48]. In these approaches, all *k*-mers of a given length receive a weight quantifying their importance defining the enhancer set. Because this *k*-mer list is typically very long, it can be difficult to interpret. Because the *k*-mer weight list has a continuous score and is logically identical to PBM data in structure, where the SVM weight is analogous to the PBM enrichment score, we anticipate that MotifSpec may be useful in summarizing important TF binding sites in weights from an SVM trained on mammalian enhancers.

In summary, we have shown that our discriminative motif finder with dynamic search spaces is comparable to or marginally outperforms all of the best existing motif discovery tools, and should improve the extraction of biologically meaningful regulatory elements from the large amounts of ChIP-seq and RNA-seq expression data being generated by high throughput sequencing technologies.

## Supporting Information

S1 Fig Q-Q plots showing fit of PBM data to log-normal distribution. Q-Q plots for two experiments, Bas1 and Pho4-9, are shown.
(PDF)

S2 Fig Effect of background model on MotifSpec ChIP-seq results. The graphs show the impact on auROC for three datasets of the order of the background model. There was very little change in auROC with the use of higher-order background models.
(PDF)

S3 Fig MotifSpec uses multiple worker processes to parallelize the motif search process. The worker threads output the motifs found, which are collected by an archiver process that creates a non-redundant archive of motifs. The motif archive is fed back into the worker processes for early termination of searches that are similar to a motif that has already been found.
(PDF)

S4 Fig Precision recall curve for CTCF. The AUC values are shown in the bottom left corner.
(PDF)

S5 Fig Precision recall curve for NRSF. The AUC values are shown in the bottom left corner.
(PDF)

S6 Fig Precision recall curve for ER. The AUC values are shown in the bottom left corner.
(PDF)

S7 Fig MotifSpec motifs predict PBM binding data better regardless of p-value threshold. The bar charts shows the improvement in the area under the receiver-operator characteristic (ROC) curve, and the top motif found by MotifSpec performs better than the Seed-and-Wobble motif regardless of the p-value threshold used to define the positive set of bound probes. The top chart shows the auROC improvement with a threshold of 0.01 and the bottom chart is with threshold 0.05.
(PDF)

S1 Table Motifs found in C. elegans by dynamic expression clustering. (PDF)
