# Supplementary figures and images for: Identification of Predictive Cis-Regulatory Elements Using a Discriminative Objective Function and a Dynamic Search Space
Source: PLoS One. 2015 Oct 14;10(10):e0140557. doi: 10.1371/journal.pone.0140557 (PMC4605740; doi:10.1371/journal.pone.0140557)

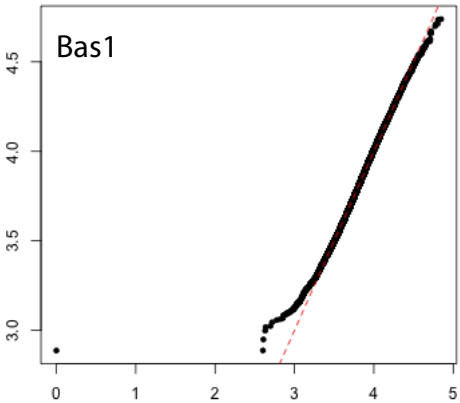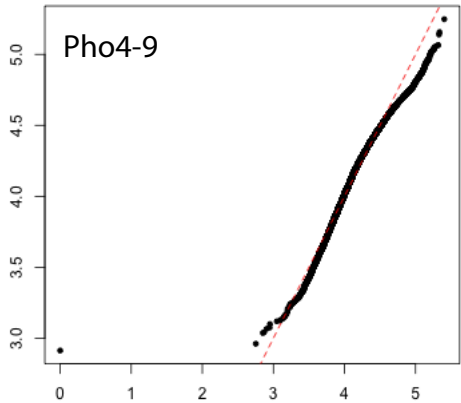

Supplement: S1 Fig — Q-Q plots for two experiments, Bas1 and Pho4-9, are shown. (PDF) [file pone.0140557.s001.pdf]

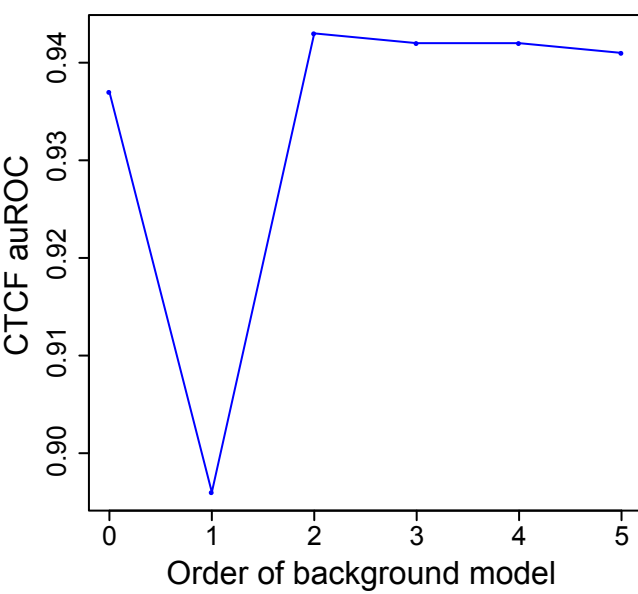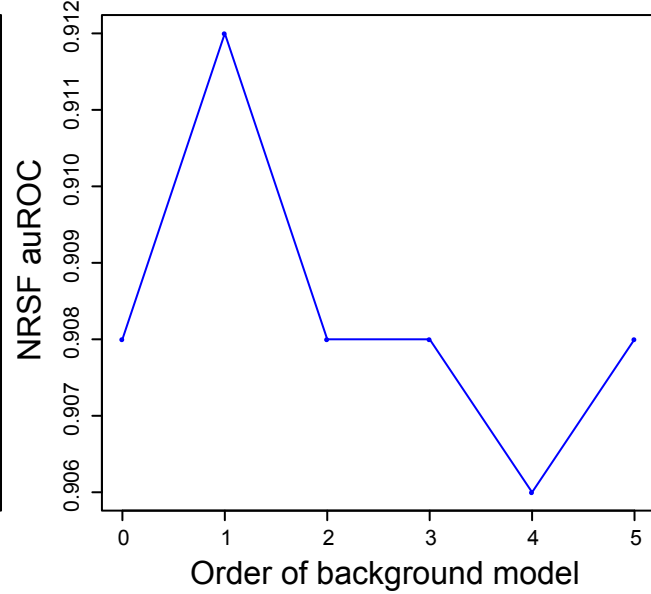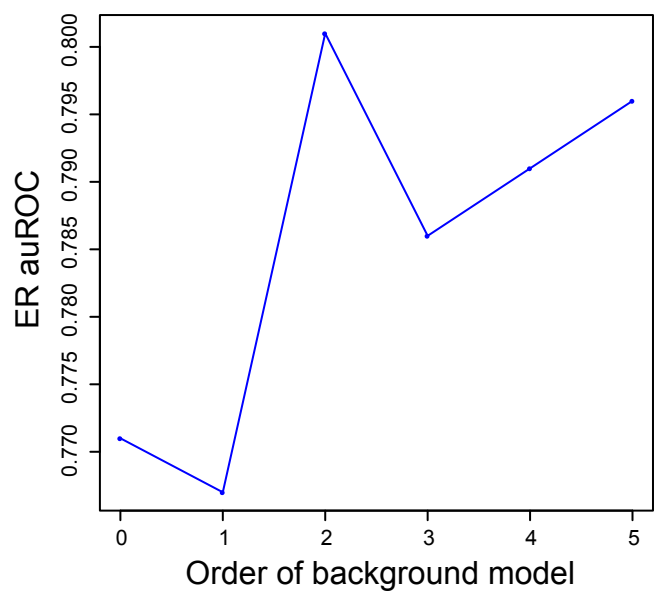

Supplement: S2 Fig — The graphs show the impact on auROC for three datasets of the order of the background model. There was very little change in auROC with the use of higher-order background models. (PDF) [file pone.0140557.s002.pdf]

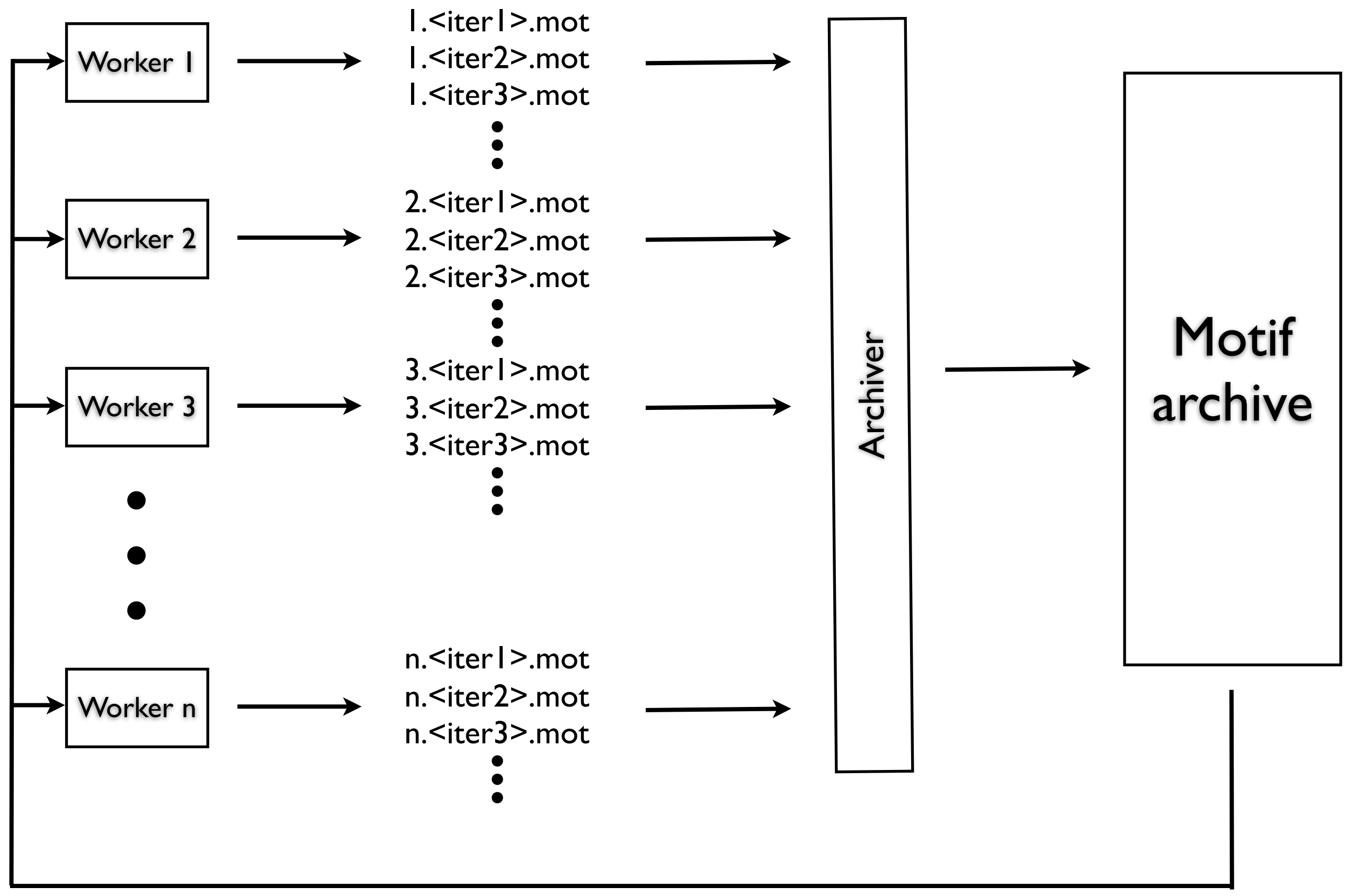

Supplement: S3 Fig — The worker threads output the motifs found, which are collected by an archiver process that creates a non-redundant archive of motifs. The motif archive is fed back into the worker processes for early termination of searches that are similar to a motif that has already been found. (PDF) [file pone.0140557.s003.pdf]

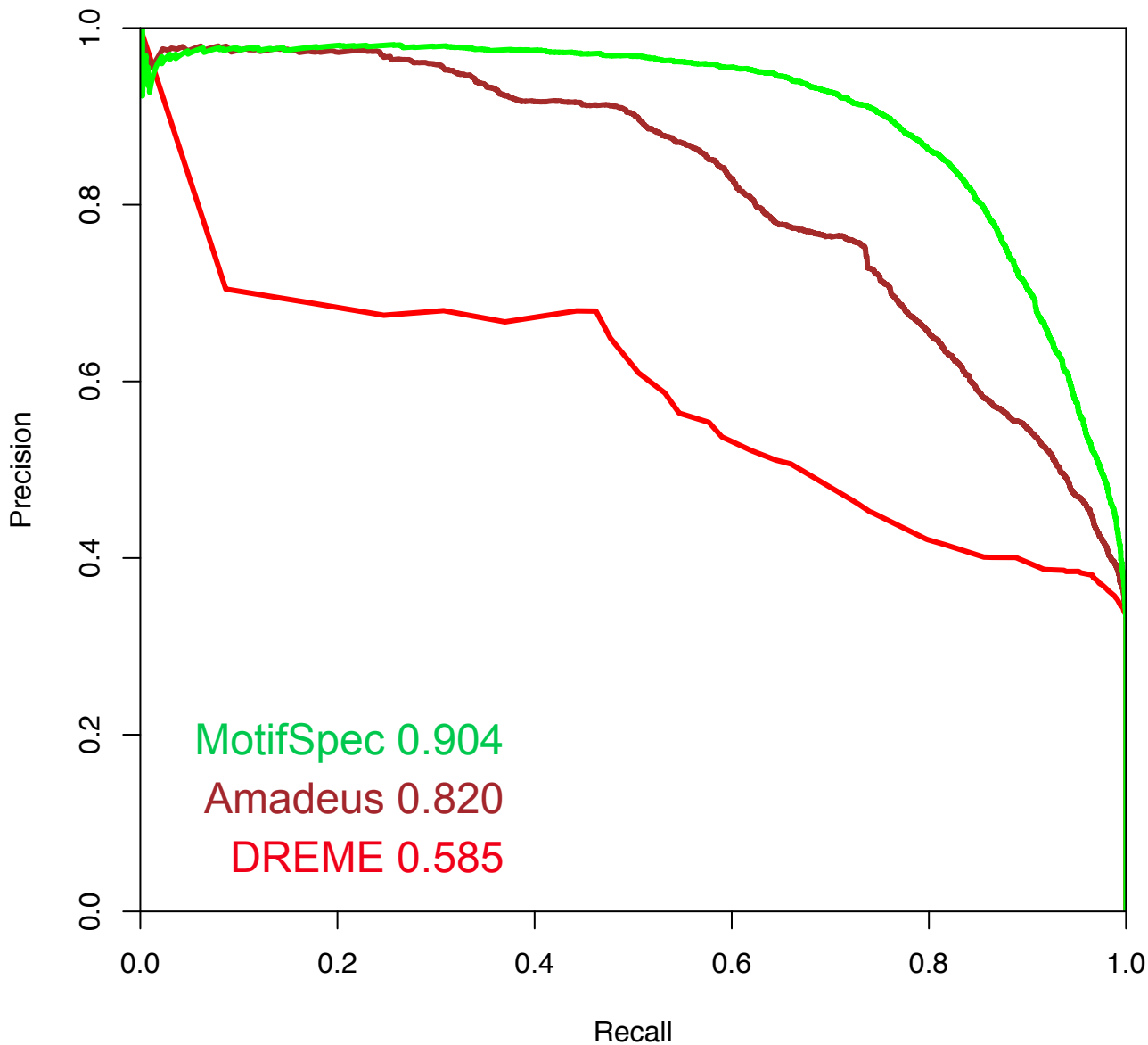

Supplement: S4 Fig — The AUC values are shown in the bottom left corner. (PDF) [file pone.0140557.s004.pdf]

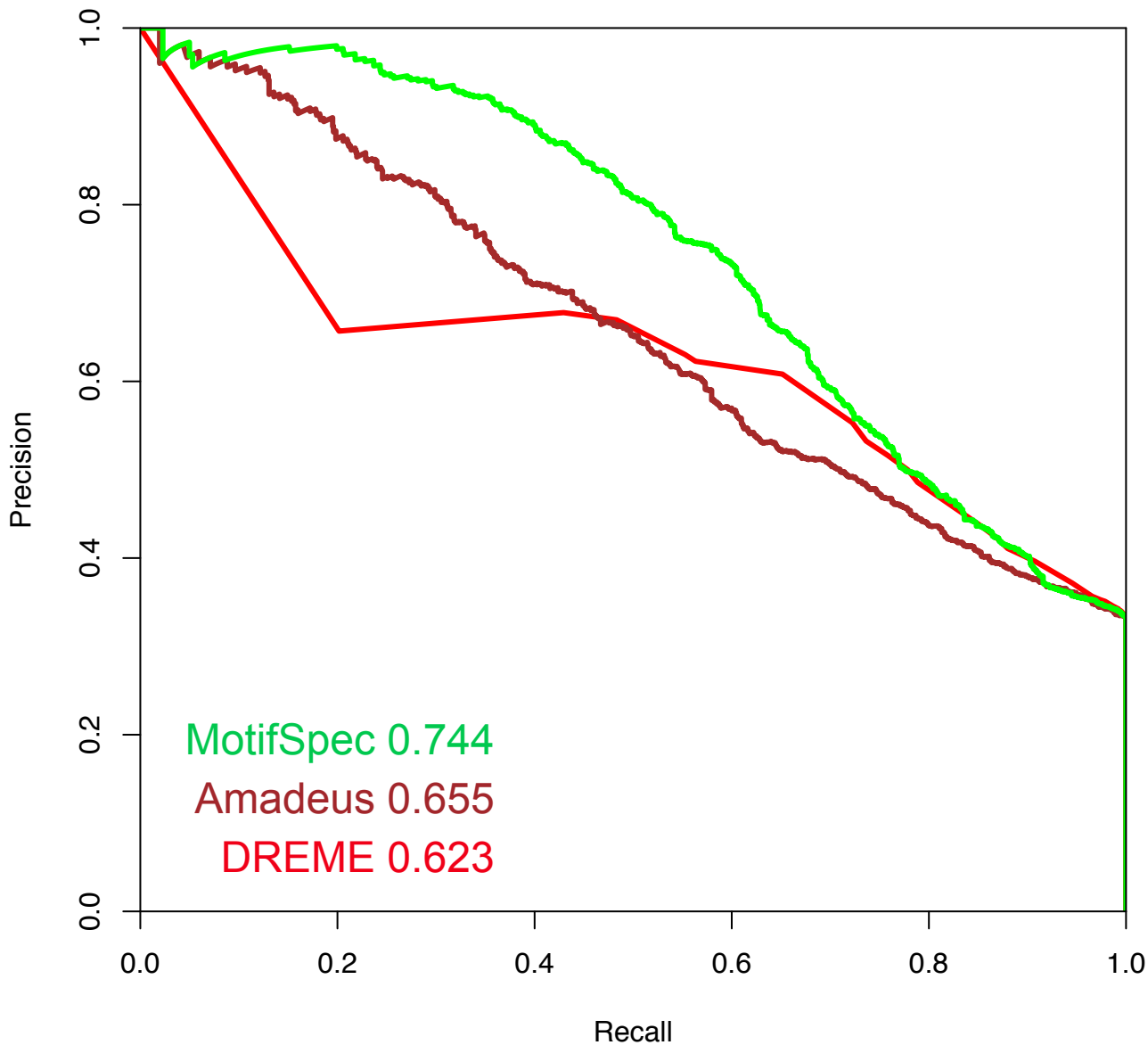

Supplement: S5 Fig — The AUC values are shown in the bottom left corner. (PDF) [file pone.0140557.s005.pdf]

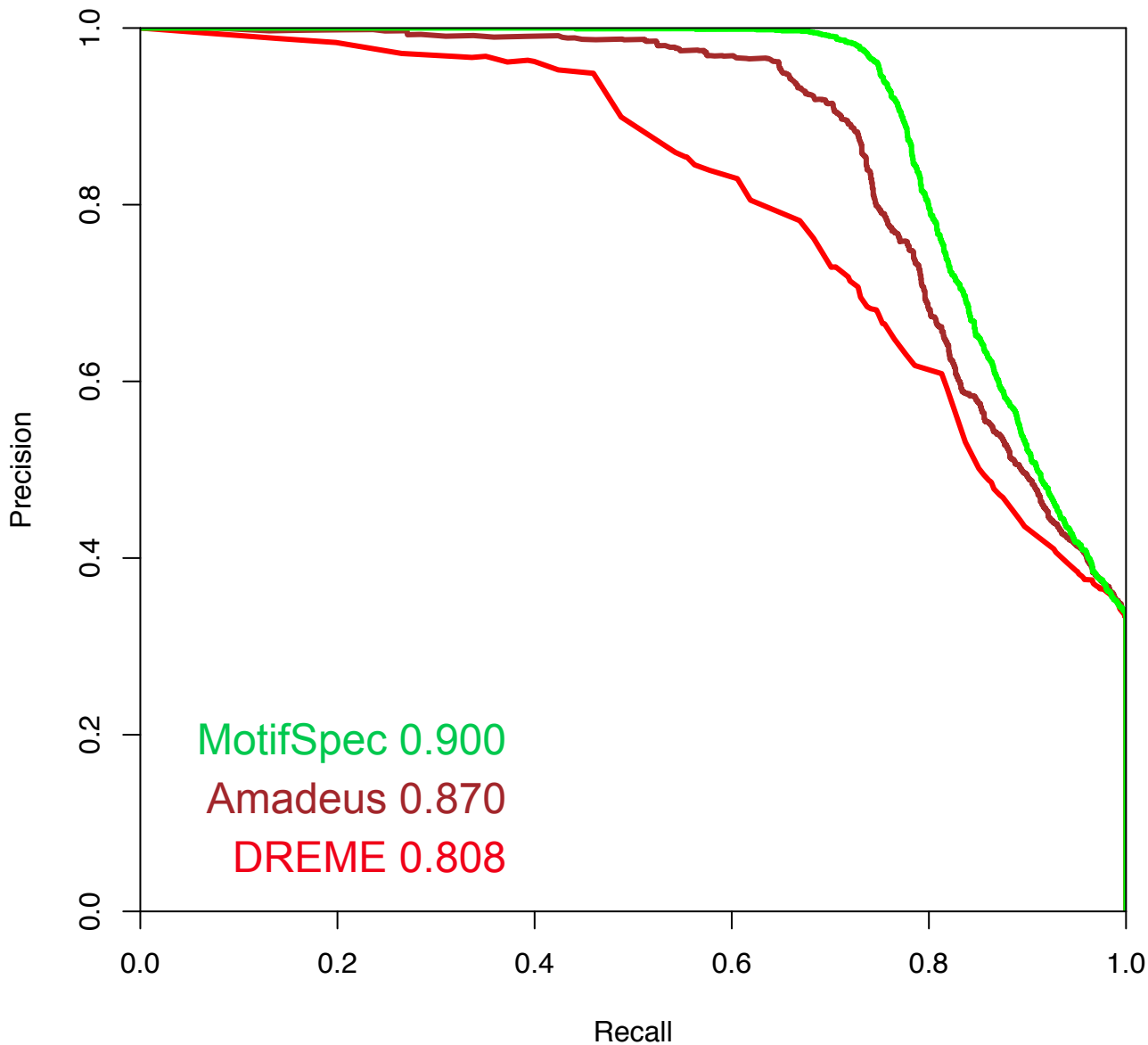

Supplement: S6 Fig — The AUC values are shown in the bottom left corner. (PDF) [file pone.0140557.s006.pdf]

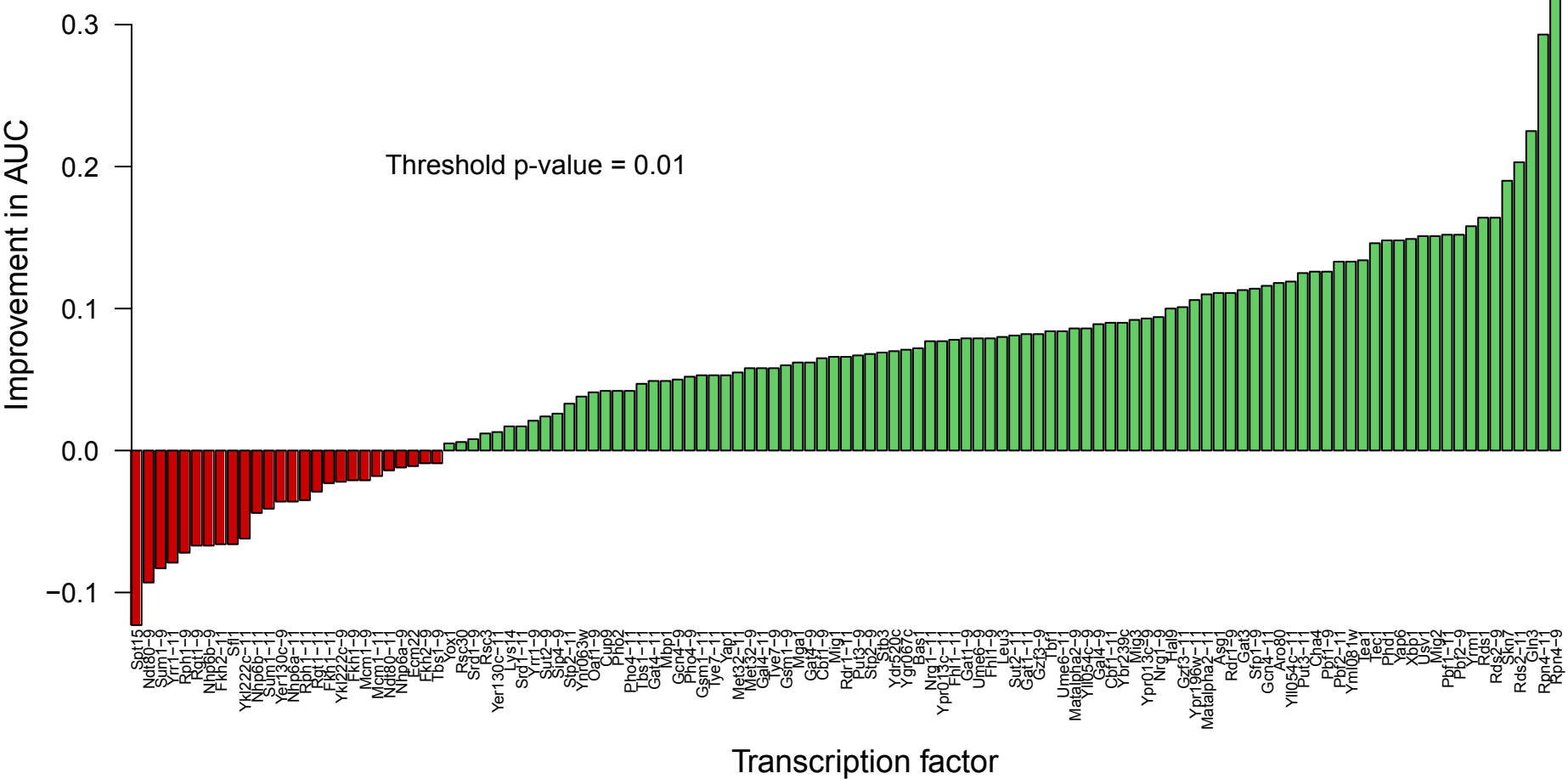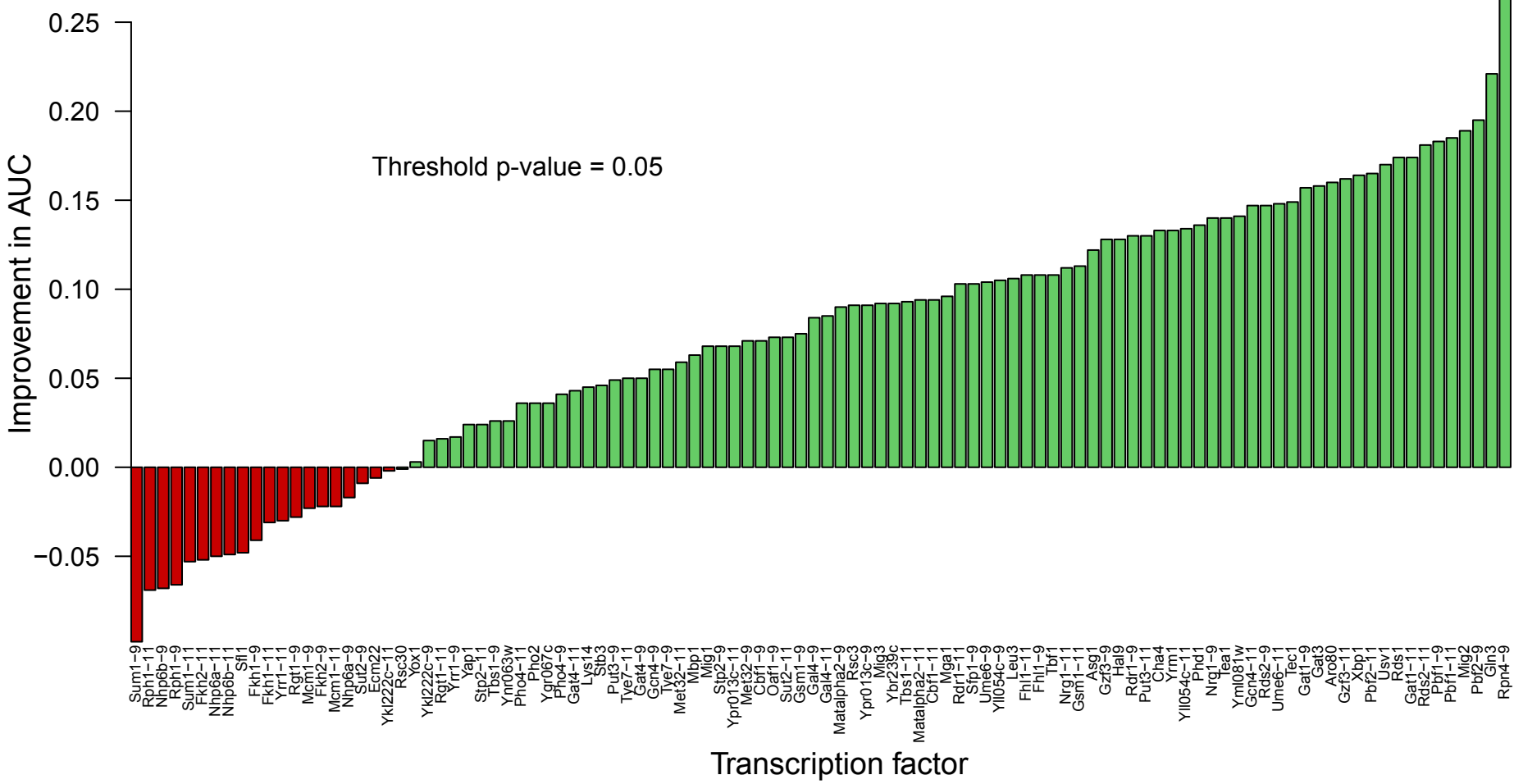

Supplement: S7 Fig — The bar charts shows the improvement in the area under the receiver-operator characteristic (ROC) curve, and the top motif found by MotifSpec performs better than the Seed-and-Wobble motif regardless of the p-value threshold used to define the positive set of bound probes. The top chart shows the auROC improvement with a threshold of 0.01 and the bottom chart is with threshold 0.05. (PDF) [file pone.0140557.s007.pdf]
