# Supplementary material for: Identification of Predictive Cis-Regulatory Elements Using a Discriminative Objective Function and a Dynamic Search Space
Source: PLoS One. 2015 Oct 14;10(10):e0140557. doi: 10.1371/journal.pone.0140557 (PMC4605740; doi:10.1371/journal.pone.0140557)
Supplement: S1 Table — (PDF) [file pone.0140557.s008.pdf]

Motif		Gene Ontology	Anatomy Ontology
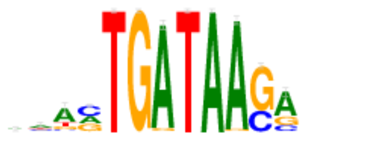	Score 83.4 Hits 465	small molecule metabolic process (2.2e-34)<br>hydrolase activity (3.5e-28)<br>biosynthetic process (5.7e-25)<br>locomotion (8.1e-22)<br>anatomical structure development (3.0e-21)	hypodermis (9.3e-04)<br>epithelial system (2.3e-03)<br>extracellular component (4.4e-03)<br>hyp8 (9.6e-03)<br>hyp9 (1.2e-02)
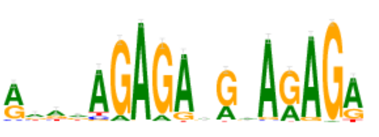	Score 81.9 Hits 480	locomotion (8.8e-59)<br>anatomical structure development (2.0e-52)<br>anatomical structure morphogenesis (9.3e-49)<br>cellular component organization (6.3e-39)<br>protein binding (8.1e-36)	muscle cell (1.2e-13)<br>hermaphrodite-specific (3.7e-13)<br>non-striated muscle (3.4e-12)<br>somatic nervous system (1.1e-11)<br>hermaphrodite gonad (4.6e-11)
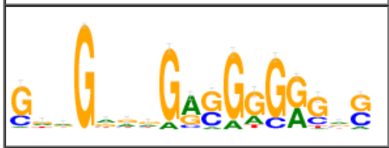	Score 65.4 Hits 345	locomotion (1.5e-47)<br>anatomical structure development (3.0e-33)<br>anatomical structure morphogenesis (6.7e-25)<br>cellular component organization (4.8e-23)<br>regulation of cellular process (5.1e-23)	ganglion (3.3e-09)<br>somatic nervous system (1.1e-08)<br>head neuron (4.7e-08)<br>muscle cell (6.0e-08)<br>epithelial system (1.2e-07)
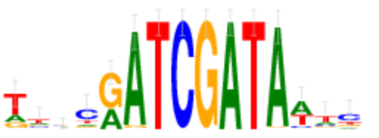	Score 62.2 Hits 48	protein binding (4.7e-05)<br>ubiquitin-dependent protein catabol (8.8e-05)<br>modification-dependent macromolecul (8.8e-05)<br>modification-dependent protein cata (8.8e-05)<br>proteolysis involved in cellular pr (1.2e-04)	
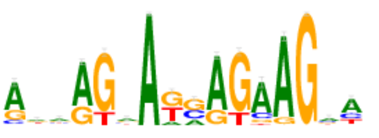	Score 60.0 Hits 529	anatomical structure morphogenesis (4.1e-44)<br>anatomical structure development (7.8e-44)<br>locomotion (2.0e-36)<br>cellular component organization (2.8e-27)<br>response to stimulus (1.5e-25)	epithelial cell (1.0e-07)<br>epithelial system (9.5e-07)<br>hypodermal cell (9.8e-07)<br>hypodermis (1.0e-06)<br>head neuron (2.4e-06)
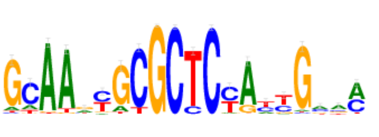	Score 59.3 Hits 494	anatomical structure development (1.2e-64)<br>reproductive process (1.8e-63)<br>organ development (1.7e-60)<br>system development (7.1e-60)<br>reproductive developmental process (1.2e-56)	Tissue (1.2e-02)<br>germ line (1.2e-02)<br>P6.p (3.8e-02)<br>P3.p (4.1e-02)<br>P4.p (4.1e-02)
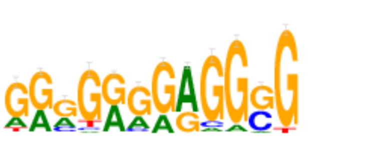	Score 54.6 Hits 404	locomotion (6.9e-41)<br>protein binding (1.2e-36)<br>intracellular organelle (1.0e-34)<br>organelle (1.2e-34)<br>anatomical structure development (5.9e-34)	hermaphrodite-specific (3.8e-12)<br>muscle cell (1.6e-10)<br>somatic neuron (6.9e-09)<br>somatic nervous system (8.8e-09)<br>body wall musculature (9.0e-09)
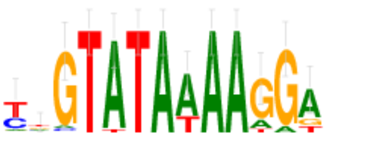	Score 53.4 Hits 23	structural constituent of cuticle (1.6e-24)<br>structural molecule activity (1.8e-20)<br>lipid transporter activity (8.1e-08)<br>lipid transport (1.5e-07)<br>alae of collagen and cuticulin-base (4.9e-04)	spermatheca (8.0e-02)
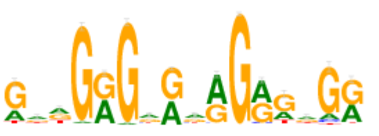	Score 51.8 Hits 547	locomotion (6.0e-59)<br>anatomical structure development (1.1e-49)<br>anatomical structure morphogenesis (7.3e-43)<br>protein binding (4.7e-35)<br>system development (2.7e-33)	epithelial cell (3.3e-11)<br>ganglion (2.3e-10)<br>hermaphrodite-specific (3.1e-10)<br>head neuron (6.7e-10)<br>epithelial system (7.9e-10)
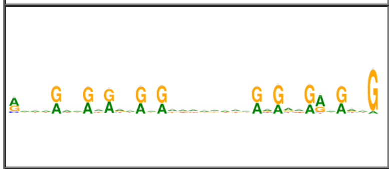	Score 39.9 Hits 938	locomotion (3.5e-82)<br>anatomical structure development (1.5e-70)<br>cytoplasm (4.6e-68)<br>growth (2.4e-60)<br>anatomical structure morphogenesis (3.2e-59)	epithelial system (5.6e-12)<br>hypodermis (1.3e-11)<br>epithelial cell (2.2e-11)<br>muscle cell (2.6e-11)<br>hermaphrodite-specific (4.3e-11)
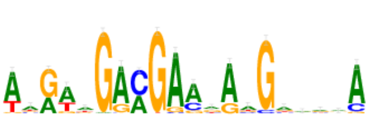	Score 38.8 Hits 311	locomotion (2.1e-40)<br>anatomical structure development (9.1e-29)<br>anatomical structure morphogenesis (4.4e-28)<br>cytoplasm (1.7e-24)<br>protein binding (9.8e-24)	body wall musculature (2.8e-09)<br>hypodermis (2.0e-07)<br>epithelial system (2.1e-07)<br>hermaphrodite gonad (2.2e-07)<br>tail (4.3e-07)
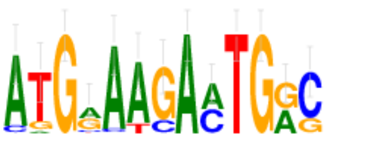	Score 38.5 Hits 19	ubiquitin-dependent protein catabol (1.8e-10)<br>modification-dependent macromolecul (1.8e-10)<br>modification-dependent protein cata (1.8e-10)<br>proteolysis involved in cellular pr (3.0e-10)<br>cellular protein catabolic process (3.1e-10)	

Motif		Gene Ontology	Anatomy Ontology
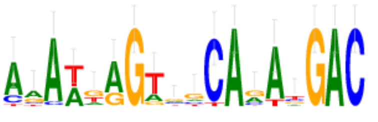	Score 37.6 Hits 17	reproductive process in a multicell (6.1e-05)<br>multicellular organism reproduction (6.2e-05)<br>spermatogenesis (8.5e-05)<br>male gamete generation (8.5e-05)<br>anatomical structure morphogenesis (9.1e-05)	
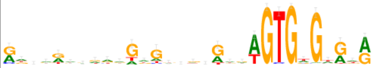	Score 37.2 Hits 149	anatomical structure development (5.6e-22)<br>locomotion (1.3e-20)<br>protein binding (1.1e-15)<br>anatomical structure morphogenesis (3.0e-15)<br>cellular component organization (9.9e-15)	epithelial cell (5.4e-05)<br>syncytium (4.2e-04)<br>vulva (4.3e-04)<br>midbody (4.3e-04)<br>epithelial system (4.5e-04)
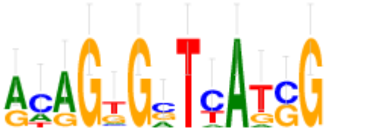	Score 37.0 Hits 11	enzyme binding (1.1e-04)<br>receptor signaling protein serine/t (2.3e-04)<br>regulation of insulin-like growth f (2.3e-04)<br>regulation of lipid storage (4.6e-04)<br>hindgut morphogenesis (7.0e-04)	blast cell (8.2e-03)<br>midbody (2.9e-02)<br>vulva (2.9e-02)<br>gonad (7.3e-02)<br>reproductive tract (7.3e-02)
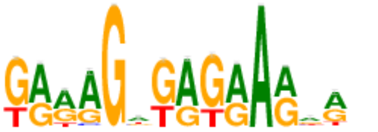	Score 35.9 Hits 1119	locomotion (8.6e-80)<br>anatomical structure development (1.5e-73)<br>anatomical structure morphogenesis (3.8e-57)<br>cytoplasm (4.0e-54)<br>growth (6.4e-54)	hypodermis (1.5e-13)<br>epithelial system (1.3e-12)<br>tail (3.1e-10)<br>muscle cell (6.6e-10)<br>ganglion (1.4e-09)
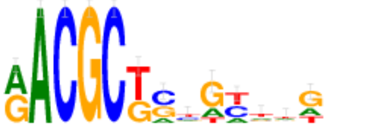	Score 35.4 Hits 84	protein binding (7.3e-08)<br>protein catabolic process (8.5e-08)<br>modification-dependent protein cata (4.3e-07)<br>ubiquitin-dependent protein catabol (4.3e-07)<br>modification-dependent macromolecul (4.3e-07)	vulE (2.4e-02)<br>vulF (2.5e-02)<br>Cpp (2.8e-02)<br>Cpa (2.8e-02)<br>Caa (2.8e-02)
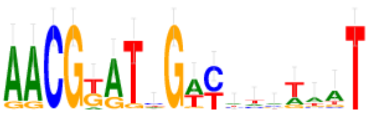	Score 35.1 Hits 23	siRNA binding (4.9e-04)<br>RNA import into nucleus (4.9e-04)<br>(1.9e-03)<br>nucleus (2.1e-03)<br>RNA transport (2.4e-03)	
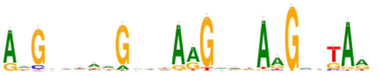	Score 34.5 Hits 440	locomotion (1.7e-40)<br>anatomical structure development (3.0e-38)<br>anatomical structure morphogenesis (3.0e-27)<br>regulation of multicellular organis (7.6e-23)<br>cytoplasm (1.4e-21)	muscle cell (3.2e-07)<br>epithelial system (2.9e-06)<br>epithelial cell (7.0e-06)<br>alimentary muscle (8.9e-06)<br>tail (2.0e-05)
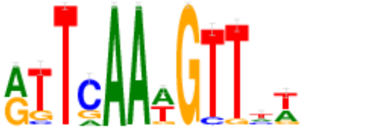	Score 34.1 Hits 369	locomotion (1.6e-27)<br>transport (1.8e-27)<br>growth (7.0e-24)<br>anatomical structure development (1.4e-23)<br>small molecule metabolic process (2.4e-23)	rectum (8.4e-05)<br>accessory cell (3.2e-04)<br>socket cell (3.3e-04)<br>interfacial epithelial cell (4.9e-04)<br>neuronal sheath cell (5.0e-04)
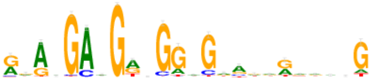	Score 33.8 Hits 837	anatomical structure development (5.3e-73)<br>locomotion (2.5e-69)<br>anatomical structure morphogenesis (2.5e-61)<br>organelle (2.4e-55)<br>intracellular organelle (9.7e-55)	somatic nervous system (1.1e-11)<br>somatic neuron (1.3e-11)<br>cholinergic neuron (1.7e-11)<br>epithelial system (2.3e-11)<br>ganglion (3.0e-11)
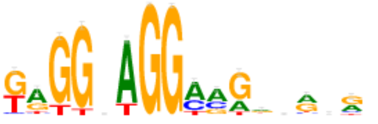	Score 33.2 Hits 660	anatomical structure development (4.0e-54)<br>anatomical structure morphogenesis (9.9e-51)<br>locomotion (3.3e-48)<br>protein binding (1.6e-41)<br>intracellular organelle (2.8e-40)	epithelial system (1.5e-08)<br>muscle cell (4.0e-08)<br>hypodermis (8.6e-08)<br>epithelial cell (1.4e-07)<br>hypodermal cell (2.8e-06)
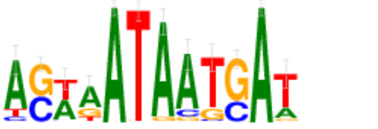	Score 32.8 Hits 88	peptidase activity, acting on L-ami (1.2e-10)<br>peptidase activity (2.1e-10)<br>hydrolase activity (4.1e-10)<br>proteolysis (9.4e-10)<br>transport (1.8e-08)	intestinal cell (3.0e-02)<br>excretory cell (4.8e-02)<br>rectum (5.1e-02)<br>rectal gland cell (5.8e-02)<br>excretory system (6.3e-02)
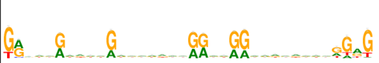	Score 32.2 Hits 896	locomotion (2.5e-68)<br>cytoplasm (1.7e-52)<br>anatomical structure development (1.3e-51)<br>anatomical structure morphogenesis (4.6e-42)<br>organelle (3.8e-39)	somatic neuron (4.9e-10)<br>tail (1.0e-09)<br>epithelial cell (1.3e-09)<br>hypodermis (1.4e-09)<br>epithelial system (1.5e-09)

Motif		Gene Ontology	Anatomy Ontology
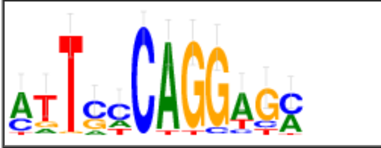	Score 32.1 Hits 28	protein binding (3.9e-08)<br>modification-dependent protein cata (1.7e-05)<br>ubiquitin-dependent protein catabol (1.7e-05)<br>modification-dependent macromolecul (1.7e-05)<br>proteolysis involved in cellular pr (2.3e-05)	Cpp (3.3e-03)<br>Cpa (3.3e-03)<br>Caa (3.3e-03)<br>Cap (3.3e-03)<br>Psub4 (5.7e-03)
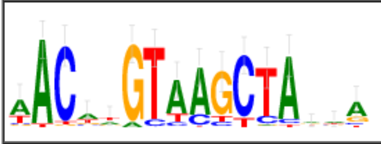	Score 32.0 Hits 42	regulation of cellular process (1.4e-05)<br>response to chemical stimulus (2.4e-05)<br>positive regulation of cellular pro (3.2e-04)<br>growth (7.2e-04)<br>regulation of cellular metabolic pr (8.3e-04)	PHCL (6.8e-04)<br>PHCR (6.8e-04)<br>uv1 (2.4e-03)<br>uterine-vulval cell (2.4e-03)<br>R4BL (8.1e-03)
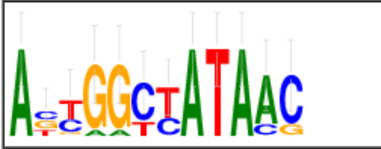	Score 32.0 Hits 10	DNA binding (2.6e-05)<br>nucleic acid binding (1.4e-04)<br>nucleosome assembly (1.5e-04)<br>nucleosome organization (1.5e-04)<br>chromatin assembly (1.5e-04)	embryonic cell (5.8e-02)<br>ganglion (8.6e-02)
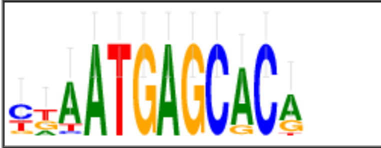	Score 32.0 Hits 13	ubiquitin-dependent protein catabol (2.4e-04)<br>modification-dependent macromolecul (2.4e-04)<br>modification-dependent protein cata (2.4e-04)<br>proteolysis involved in cellular pr (2.9e-04)<br>cellular protein catabolic process (3.0e-04)	
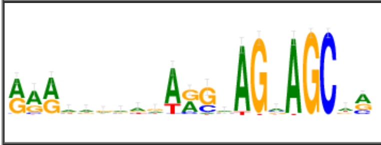	Score 31.8 Hits 122	anatomical structure development (1.5e-17)<br>locomotion (1.0e-15)<br>anatomical structure morphogenesis (8.3e-13)<br>system development (1.8e-12)<br>cellular component organization (1.1e-11)	ganglion (4.1e-06)<br>muscle cell (6.7e-06)<br>somatic nervous system (9.3e-06)<br>tail neuron (1.8e-05)<br>tail ganglion (2.5e-05)
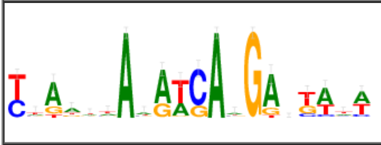	Score 31.8 Hits 101	heterocycle metabolic process (2.3e-06)<br>negative regulation of response to (3.4e-06)<br>melanin metabolic process (4.5e-06)<br>ammonia-lyase activity (4.5e-06)<br>melanin biosynthetic process (4.5e-06)	lateral ganglion (5.9e-08)<br>lateral pharyngeal ganglion right n (2.2e-07)<br>lateral pharyngeal ganglion left (2.2e-07)<br>lateral pharyngeal ganglion left ne (2.2e-07)<br>lateral pharyngeal ganglion right (2.2e-07)
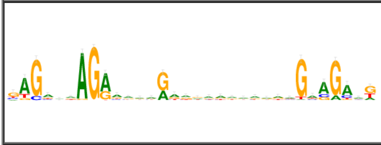	Score 31.8 Hits 285	locomotion (2.4e-36)<br>anatomical structure development (2.4e-21)<br>intracellular organelle (2.5e-21)<br>organelle (2.9e-21)<br>growth (3.9e-21)	hermaphrodite-specific (4.6e-07)<br>muscle cell (6.6e-06)<br>non-striated muscle (6.7e-06)<br>body muscle cell (1.4e-05)<br>pharynx (1.6e-05)
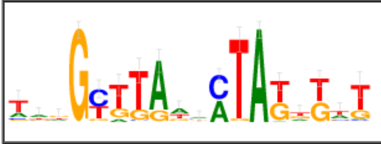	Score 31.8 Hits 62	locomotion (2.0e-11)<br>molting cycle (1.2e-08)<br>molting cycle, collagen and cuticul (1.2e-08)<br>molting cycle, protein-based cuticl (1.2e-08)<br>anatomical structure development (1.9e-08)	hyp7 syncytium (3.0e-04)<br>hypodermal cell (1.6e-03)<br>seam cell (5.4e-03)<br>hyp12 (1.1e-02)<br>epithelial cell (1.3e-02)
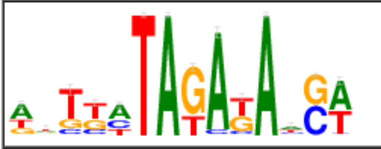	Score 31.4 Hits 433	hydrolase activity (1.3e-28)<br>locomotion (3.3e-27)<br>cytoplasm (6.7e-22)<br>anatomical structure development (4.6e-21)<br>anatomical structure morphogenesis (1.0e-18)	epithelial system (2.2e-04)<br>hyp8 (2.2e-04)<br>epithelial cell (2.7e-04)<br>hyp9 (3.0e-04)<br>hyp6 (8.8e-04)
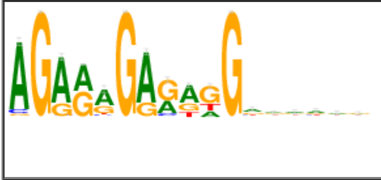	Score 30.9 Hits 753	cytoplasm (4.2e-52)<br>anatomical structure development (3.2e-50)<br>locomotion (6.9e-50)<br>anatomical structure morphogenesis (3.4e-48)<br>intracellular organelle (6.6e-48)	muscle cell (1.1e-10)<br>body wall musculature (2.3e-10)<br>non-striated muscle (1.1e-09)<br>body muscle cell (1.9e-09)<br>hermaphrodite-specific (4.5e-09)
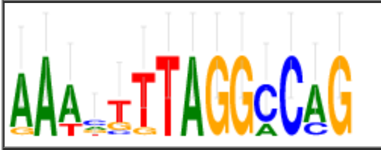	Score 30.9 Hits 8	structural constituent of ribosome (5.0e-21)<br>ribosome (5.0e-21)<br>ribonucleoprotein complex (3.9e-20)<br>translation (3.6e-19)<br>gene expression (6.7e-17)	
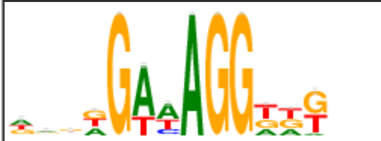	Score 30.8 Hits 738	locomotion (3.4e-46)<br>transport (5.7e-38)<br>anatomical structure development (3.0e-36)<br>cytoplasm (9.4e-36)<br>anatomical structure morphogenesis (2.3e-34)	muscle cell (1.7e-06)<br>non-striated muscle (2.1e-05)<br>interneuron (4.0e-05)<br>hypodermis (4.6e-05)<br>epithelial system (5.6e-05)

Motif		Gene Ontology	Anatomy Ontology
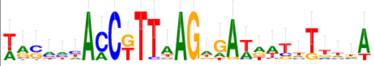	Score 30.7 Hits 11	ubiquitin-dependent protein catabol (3.0e-09)<br>modification-dependent macromolecul (3.0e-09)<br>modification-dependent protein cata (3.0e-09)<br>proteolysis involved in cellular pr (4.5e-09)<br>cellular protein catabolic process (4.8e-09)	
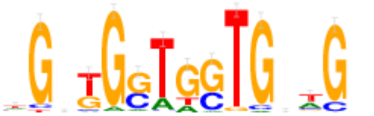	Score 30.3 Hits 288	locomotion (6.1e-45)<br>anatomical structure morphogenesis (1.0e-32)<br>anatomical structure development (1.3e-32)<br>cellular component organization (1.1e-23)<br>protein binding (1.1e-22)	muscle cell (5.5e-11)<br>non-striated muscle (8.1e-10)<br>body muscle cell (2.0e-08)<br>ganglion (2.5e-08)<br>epithelial system (7.8e-08)
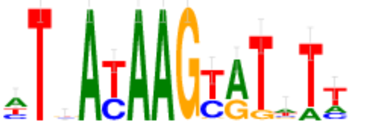	Score 29.8 Hits 57	cytoplasm (1.5e-06)<br>serine-type peptidase activity (2.3e-06)<br>serine hydrolase activity (2.3e-06)<br>peptidase activity, acting on L-ami (5.9e-06)<br>small molecule metabolic process (6.8e-06)	hypodermis (6.2e-02)<br>epithelial system (8.8e-02)
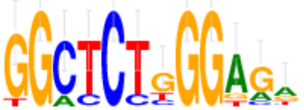	Score 29.8 Hits 16	regulation of macromolecule biosynt (1.2e-04)<br>regulation of cellular biosynthetic (1.2e-04)<br>regulation of biosynthetic process (1.2e-04)<br>regulation of cellular metabolic pr (1.5e-04)<br>regulation of primary metabolic pro (1.5e-04)	hypodermis (2.5e-03)<br>epithelial system (3.5e-03)<br>ventral nerve cord (8.7e-03)<br>head neuron (6.6e-02)<br>midbody (7.7e-02)
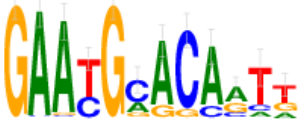	Score 29.7 Hits 60	cellular macromolecule metabolic pr (7.0e-09)<br>macromolecule metabolic process (1.9e-08)<br>anatomical structure development (9.0e-08)<br>anatomical structure morphogenesis (1.2e-07)<br>cellular component organization (4.8e-07)	gland cell (5.8e-02)
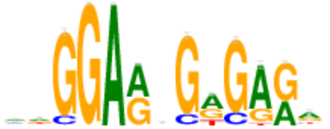	Score 29.5 Hits 961	anatomical structure development (8.3e-69)<br>locomotion (7.5e-68)<br>growth (4.2e-58)<br>anatomical structure morphogenesis (4.5e-57)<br>cytoplasm (3.7e-53)	non-striated muscle (1.4e-12)<br>body muscle cell (1.4e-12)<br>muscle cell (4.6e-12)<br>hermaphrodite-specific (9.3e-11)<br>vulval muscle (1.0e-10)
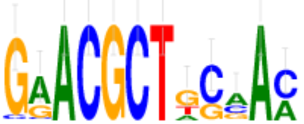	Score 29.5 Hits 17	ubiquitin-dependent protein catabol (2.2e-08)<br>modification-dependent macromolecul (2.2e-08)<br>modification-dependent protein cata (2.2e-08)<br>proteolysis involved in cellular pr (3.3e-08)<br>cellular protein catabolic process (3.4e-08)	
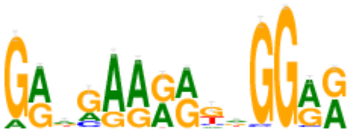	Score 29.4 Hits 473	locomotion (8.6e-54)<br>anatomical structure development (8.2e-43)<br>anatomical structure morphogenesis (4.5e-41)<br>growth (1.2e-30)<br>cytoplasm (2.4e-29)	muscle cell (1.1e-08)<br>epithelial system (3.6e-07)<br>non-striated muscle (4.0e-07)<br>hypodermis (5.2e-07)<br>body muscle cell (3.5e-06)
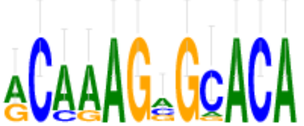	Score 29.4 Hits 10	phagocytic cup (2.1e-04)<br>midbody (1.1e-03)<br>phagocytic vesicle (1.1e-03)<br>pseudopodium (1.9e-03)<br>cell division site part (2.3e-03)	non-striated muscle (2.1e-02)<br>muscle cell (2.6e-02)<br>pharyngeal muscle cell (2.9e-02)<br>body muscle cell (5.9e-02)<br>seam cell (6.9e-02)
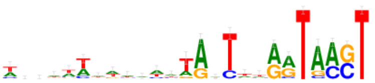	Score 29.4 Hits 111	metal ion binding (1.7e-06)<br>cation binding (3.0e-06)<br>ion binding (3.0e-06)<br>melanin metabolic process (5.4e-06)<br>melanin biosynthetic process (5.4e-06)	interneuron (1.2e-06)<br>lateral pharyngeal ganglion right n (4.5e-06)<br>lateral pharyngeal ganglion left (4.5e-06)<br>lateral pharyngeal ganglion left ne (4.5e-06)<br>lateral pharyngeal ganglion right (4.5e-06)
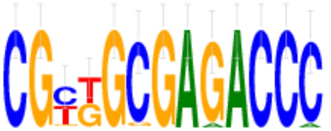	Score 29.2 Hits 19	structural constituent of ribosome (2.3e-35)<br>ribosome (2.3e-35)<br>ribonucleoprotein complex (1.1e-33)<br>translation (8.0e-32)<br>non-membrane-bounded organelle (1.1e-28)	
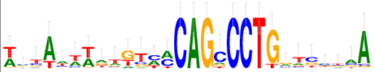	Score 29.2 Hits 13	ubiquitin-dependent protein catabol (1.5e-06)<br>modification-dependent macromolecul (1.5e-06)<br>modification-dependent protein cata (1.5e-06)<br>proteolysis involved in cellular pr (2.1e-06)<br>cellular protein catabolic process (2.1e-06)	

Motif		Gene Ontology	Anatomy Ontology
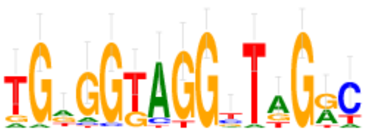	Score 29.1 Hits 12	regulation of neuron differentiatio (2.2e-05)<br>regulation of neurogenesis (2.2e-05)<br>regulation of cell development (2.4e-05)<br>regulation of nervous system develo (2.9e-05)<br>regulation of cell differentiation (9.7e-05)	Caapa (3.2e-04)<br>ABplapppapp (3.7e-04)<br>ABplapppaap (3.7e-04)<br>ABplapppaa (3.7e-04)<br>ABprapppapa (3.7e-04)
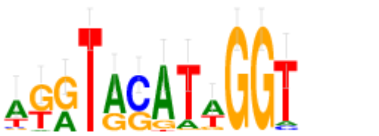	Score 29.0 Hits 25	plasma membrane (6.3e-07)<br>protein binding (3.7e-06)<br>anatomical structure development (4.1e-06)<br>anatomical structure morphogenesis (4.9e-06)<br>cell-cell adherens junction (1.5e-05)	blast cell (3.8e-03)<br>ray 3 left (4.6e-03)<br>ray 3 (4.6e-03)<br>ray 3 right (4.6e-03)<br>ray 7 (4.9e-03)
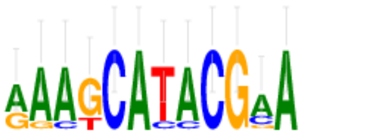	Score 28.9 Hits 8	somatic stem cell division (1.7e-04)<br>neuroblast division (1.7e-04)<br>neuroblast proliferation (1.7e-04)<br>vulval location (8.4e-04)<br>cell proliferation (1.2e-03)	ventral cord neuron (8.2e-02)
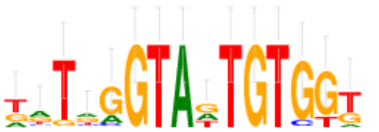	Score 28.9 Hits 9	protein binding (1.8e-06)<br>behavior (9.7e-05)<br>regulation of localization (1.4e-04)<br>reproductive process in a multicell (1.8e-04)<br>multicellular organism reproduction (1.8e-04)	procorpus (7.7e-04)<br>metacorpus (2.8e-03)<br>corpus (3.1e-03)<br>terminal bulb (4.9e-03)<br>pharyngeal segment (6.9e-03)
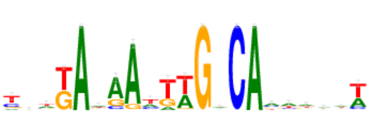	Score 28.8 Hits 319	cytoplasm (2.0e-17)<br>macromolecule metabolic process (1.3e-15)<br>protein metabolic process (2.3e-15)<br>small molecule metabolic process (6.0e-14)<br>transferase activity (1.2e-13)	sensory neuron (1.2e-04)<br>motor neuron (1.7e-04)<br>ray (3.5e-04)<br>sensillum (3.6e-04)<br>lateral ganglion (4.2e-04)
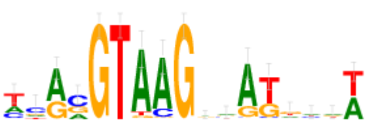	Score 28.6 Hits 46	ammonia-lyase activity (9.2e-07)<br>neuropeptide signaling pathway (2.5e-06)<br>carbon-nitrogen lyase activity (1.4e-05)<br>aromatic amino acid family metaboli (8.3e-05)<br>lyase activity (1.6e-04)	interneuron (7.9e-06)<br>amphid right sensillum (1.3e-05)<br>amphid left sensillum (1.3e-05)<br>lateral pharyngeal ganglion right n (1.6e-05)<br>lateral pharyngeal ganglion left (1.6e-05)
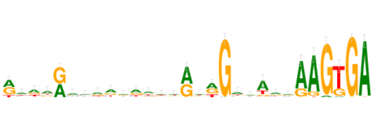	Score 28.4 Hits 85	anatomical structure development (2.5e-13)<br>protein binding (1.6e-12)<br>system development (1.1e-11)<br>metal ion binding (9.8e-11)<br>organ development (1.3e-10)	syncytium (1.1e-04)<br>epithelial system (1.4e-04)<br>hypodermis (1.5e-04)<br>preanal ganglion (2.0e-04)<br>epithelial cell (3.2e-04)
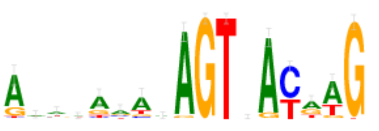	Score 28.2 Hits 137	cellular biosynthetic process (8.7e-09)<br>small molecule metabolic process (1.9e-08)<br>biosynthetic process (4.7e-08)<br>heterocycle metabolic process (5.2e-08)<br>phosphate metabolic process (5.6e-08)	pharyngeal interneuron (1.2e-05)<br>metacorpus (2.0e-04)<br>corpus (2.9e-04)<br>pharyngeal neuron (6.2e-04)<br>pharyngeal nervous system (6.2e-04)
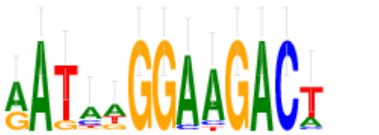	Score 28.2 Hits 12	ubiquitin-dependent protein catabol (1.2e-11)<br>modification-dependent macromolecul (1.2e-11)<br>modification-dependent protein cata (1.2e-11)<br>proteolysis involved in cellular pr (2.1e-11)<br>cellular protein catabolic process (2.2e-11)	
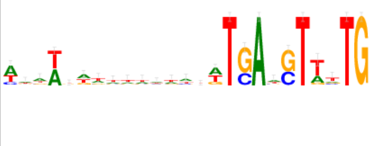	Score 28.0 Hits 86	structural constituent of cuticle (1.7e-08)<br>transport (4.4e-07)<br>apical part of cell (4.5e-07)<br>hydrolase activity (6.9e-07)<br>proteolysis (1.9e-06)	rectal gland cell (4.2e-03)<br>(5.2e-03)<br>diagonal muscle (1.2e-02)<br>gland cell (3.1e-02)
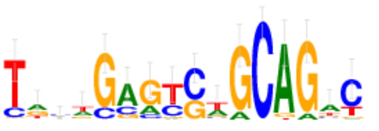	Score 28.0 Hits 31	ubiquitin-dependent protein catabol (2.5e-09)<br>modification-dependent macromolecul (2.5e-09)<br>modification-dependent protein cata (2.5e-09)<br>proteolysis involved in cellular pr (4.3e-09)<br>cellular protein catabolic process (4.5e-09)	
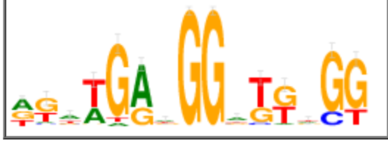	Score 27.8 Hits 197	locomotion (3.7e-31)<br>anatomical structure development (1.2e-30)<br>anatomical structure morphogenesis (2.2e-24)<br>cellular component organization (1.0e-18)<br>system development (5.7e-17)	muscle cell (6.4e-07)<br>non-striated muscle (3.3e-06)<br>hermaphrodite-specific (5.9e-06)<br>body muscle cell (9.6e-06)<br>motor neuron (1.5e-05)

Motif		Gene Ontology	Anatomy Ontology
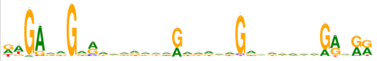	Score 27.7 Hits 625	locomotion (5.0e-62)<br>anatomical structure development (3.2e-56)<br>cytoplasm (2.5e-51)<br>intracellular organelle (2.8e-47)<br>organelle (3.6e-47)	muscle cell (2.2e-14)<br>non-striated muscle (3.6e-13)<br>epithelial system (1.8e-12)<br>hermaphrodite-specific (7.3e-12)<br>hypodermis (8.0e-12)
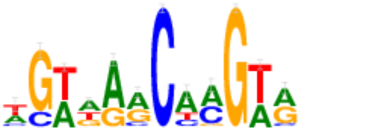	Score 27.7 Hits 152	biosynthetic process (7.8e-11)<br>small molecule metabolic process (9.0e-10)<br>oxoacid metabolic process (3.6e-09)<br>carboxylic acid metabolic process (3.6e-09)<br>organic acid metabolic process (3.6e-09)	lumbar lateral left ganglion (3.1e-04)<br>lateral pharyngeal ganglion right n (4.1e-04)<br>lateral pharyngeal ganglion left (4.1e-04)<br>lateral pharyngeal ganglion left ne (4.1e-04)<br>lateral pharyngeal ganglion right (4.1e-04)
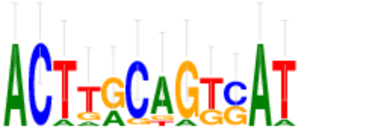	Score 27.7 Hits 8	nuclear speck (1.0e-03)<br>plasma membrane (1.2e-03)<br>nuclear body (1.4e-03)<br>amino acid transmembrane transporte (2.4e-03)<br>amino acid transport (2.7e-03)	sensillum (3.4e-02)<br>embryonic cell (3.8e-02)<br>hypodermal cell (6.5e-02)
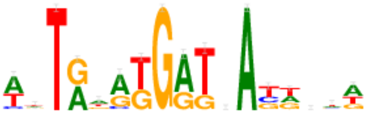	Score 27.7 Hits 177	small molecule metabolic process (1.1e-10)<br>biosynthetic process (1.6e-10)<br>metal ion binding (2.7e-10)<br>cation binding (6.9e-10)<br>ion binding (6.9e-10)	interneuron (1.1e-05)<br>sensory neuron (4.8e-05)<br>lumbar lateral right ganglion (6.9e-05)<br>lumbar lateral ganglion (6.9e-05)<br>motor neuron (7.1e-05)
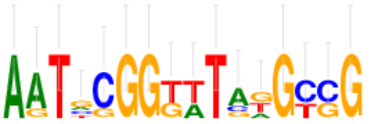	Score 27.7 Hits 5	plasma membrane (3.0e-06)<br>positive regulation of locomotion (1.3e-04)<br>regulation of locomotion (3.0e-04)<br>nuclear speck (6.3e-04)<br>nuclear body (8.4e-04)	
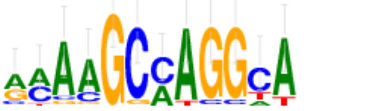	Score 27.6 Hits 14	neuroblast fate specification (8.9e-04)<br>protein heterodimerization activity (1.8e-03)<br>neuroblast fate commitment (1.8e-03)<br>neuroblast differentiation (2.1e-03)<br>DNA binding (2.2e-03)	ganglion (5.8e-02)
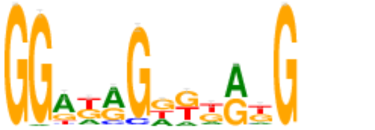	Score 27.6 Hits 787	anatomical structure development (2.5e-52)<br>locomotion (5.0e-50)<br>growth (8.0e-46)<br>anatomical structure morphogenesis (1.4e-41)<br>organelle (5.8e-40)	muscle cell (3.0e-06)<br>hypodermis (3.3e-06)<br>non-striated muscle (1.7e-05)<br>epithelial system (2.1e-05)<br>hypodermal cell (1.1e-04)
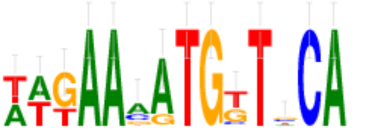	Score 27.6 Hits 16	structural constituent of cuticle (2.1e-07)<br>dopamine beta-monooxygenase activit (9.7e-06)<br>histidine catabolic process (1.5e-05)<br>histidine family amino acid metabol (1.5e-05)<br>histidine metabolic process (1.5e-05)	gonadal sheath cell (3.7e-02)<br>muscle of the reproductive system (4.3e-02)
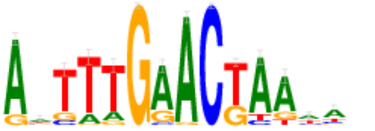	Score 27.5 Hits 382	small molecule metabolic process (8.1e-27)<br>anatomical structure development (3.6e-24)<br>biosynthetic process (4.0e-20)<br>determination of adult lifespan (2.2e-18)<br>multicellular organismal aging (2.2e-18)	spermathecal-uterine junction (4.8e-04)<br>corpus (2.9e-03)<br>syncytium (4.1e-03)<br>metacorpus (6.5e-03)<br>pharyngeal cell (1.2e-02)
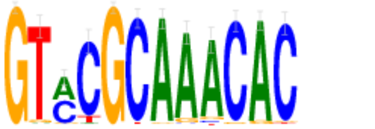	Score 27.4 Hits 162	growth (3.8e-26)<br>intracellular organelle (1.7e-20)<br>organelle (1.8e-20)<br>cellular component organization (5.3e-19)<br>anatomical structure development (5.0e-17)	gon_herm_dtc_A (9.9e-03)<br>hermaphrodite distal tip cell (1.0e-02)<br>anterior gonad arm (1.0e-02)<br>DTC (1.0e-02)<br>gon_herm_dtc_P (1.0e-02)
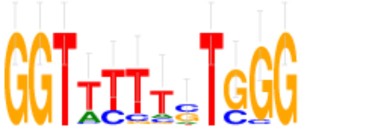	Score 27.3 Hits 13	SCF ubiquitin ligase complex (5.5e-04)<br>cell proliferation (1.9e-03)<br>cullin-RING ubiquitin ligase comple (2.5e-03)<br>post-embryonic body morphogenesis (2.7e-03)<br>protein binding (4.9e-03)	
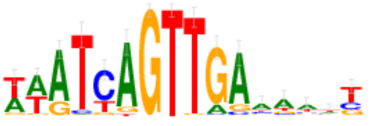	Score 27.3 Hits 103	transport (7.6e-09)<br>cellular biosynthetic process (1.5e-08)<br>biosynthetic process (6.7e-08)<br>small molecule metabolic process (7.0e-08)<br>heterocycle metabolic process (1.3e-07)	uv1 (1.4e-02)<br>uterine-vulval cell (1.4e-02)<br>rect_VL (2.6e-02)<br>rect_VR (2.6e-02)<br>uterine seam cell (2.9e-02)

Motif		Gene Ontology	Anatomy Ontology
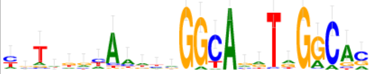	Score 27.2 Hits 22	ubiquitin-dependent protein catabol (6.6e-08)<br>modification-dependent macromolecul (6.6e-08)<br>modification-dependent protein cata (6.6e-08)<br>proteolysis involved in cellular pr (9.9e-08)<br>cellular protein catabolic process (1.0e-07)	
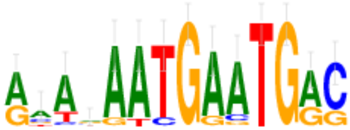	Score 27.0 Hits 28	spermatogenesis (2.3e-04)<br>male gamete generation (2.3e-04)<br>nucleus (5.2e-04)<br>gastrulation with mouth forming fir (6.4e-04)<br>(1.0e-03)	amphid socket cell (4.8e-03)<br>AMsoR (4.8e-03)<br>AMsoL (4.8e-03)<br>hyp1 (6.6e-03)<br>anterior pharyngeal ganglion (ant) (6.6e-03)
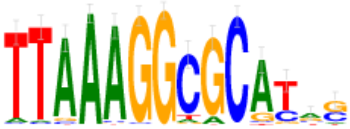	Score 27.0 Hits 128	cytoplasm (1.3e-20)<br>germ plasm (8.7e-17)<br>pole plasm (8.7e-17)<br>P granule (8.7e-17)<br>intracellular organelle (5.8e-16)	Tissue (1.7e-11)<br>germ line (1.7e-11)<br>Z2 (4.8e-04)<br>germline precursor cell (4.8e-04)<br>Z3 (4.8e-04)
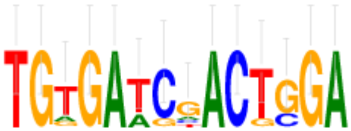	Score 27.0 Hits 7	ubiquitin-dependent protein catabol (3.2e-10)<br>modification-dependent macromolecul (3.2e-10)<br>modification-dependent protein cata (3.2e-10)<br>proteolysis involved in cellular pr (4.9e-10)<br>cellular protein catabolic process (5.1e-10)	
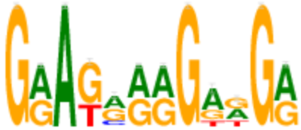	Score 26.4 Hits 321	locomotion (3.4e-31)<br>anatomical structure development (1.7e-22)<br>anatomical structure morphogenesis (1.1e-20)<br>growth (8.3e-19)<br>cytoplasm (4.2e-18)	anal depressor muscle (7.6e-07)<br>smooth muscle (2.9e-06)<br>uterine muscle (1.5e-05)<br>alimentary muscle (4.5e-05)<br>vulval muscle (6.9e-05)
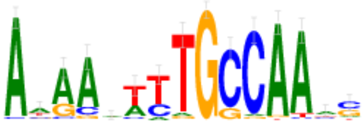	Score 26.4 Hits 121	anatomical structure morphogenesis (2.0e-09)<br>anatomical structure development (3.3e-09)<br>oxidoreductase activity, acting on (8.9e-09)<br>oxidoreductase activity, acting on (1.2e-08)<br>cation binding (7.3e-08)	CEP (2.9e-03)<br>excretory system (9.0e-03)<br>tail hypodermis (9.2e-03)<br>excretory secretory system (1.4e-02)<br>excretory cell (1.6e-02)
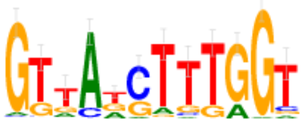	Score 26.4 Hits 96	anatomical structure development (2.1e-10)<br>growth (2.2e-10)<br>small molecule metabolic process (2.9e-09)<br>cellular biosynthetic process (6.0e-08)<br>transport (7.5e-08)	hmc (5.2e-03)<br>intestinal cell (3.7e-02)<br>uterine muscle (5.3e-02)<br>excretory cell (7.1e-02)<br>rectum (7.8e-02)
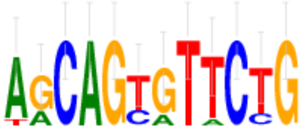	Score 26.3 Hits 10	male gamete generation (2.8e-05)<br>spermatogenesis (2.8e-05)<br>gastrulation with mouth forming fir (7.8e-05)<br>protein binding (1.1e-04)<br>(1.3e-04)	
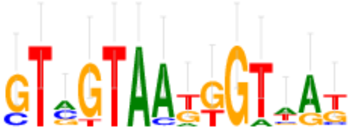	Score 26.2 Hits 8	axonal defasciculation (1.7e-04)<br>(3.4e-04)<br>endodeoxyribonuclease activity, pro (5.1e-04)<br>deoxyribonuclease II activity (5.1e-04)<br>endonuclease activity, active with (1.0e-03)	epithelial system (8.7e-02)
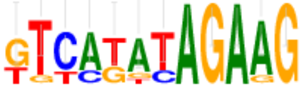	Score 26.2 Hits 8	unfolded protein binding (7.1e-03)<br>ATP binding (9.7e-03)<br>adenyl ribonucleotide binding (9.7e-03)<br>adenyl nucleotide binding (1.1e-02)<br>purine nucleoside binding (1.1e-02)	
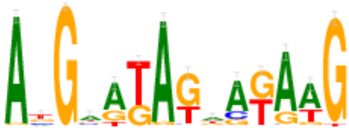	Score 26.1 Hits 270	anatomical structure development (3.0e-17)<br>anatomical structure morphogenesis (8.6e-16)<br>locomotion (1.9e-13)<br>transport (3.2e-12)<br>regulation of biological quality (9.2e-12)	epithelial system (2.5e-05)<br>epithelial cell (4.4e-05)<br>extracellular component (2.2e-04)<br>hypodermis (2.3e-04)<br>interfacial epithelial cell (5.0e-04)
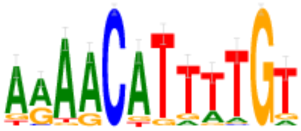	Score 26.1 Hits 159	transporter activity (1.9e-12)<br>small molecule metabolic process (1.9e-10)<br>transmembrane transporter activity (3.0e-10)<br>hydrolase activity (4.2e-10)<br>transport (2.3e-09)	excretory cell (1.7e-02)<br>excretory system (2.6e-02)<br>excretory secretory system (3.8e-02)

Motif		Gene Ontology	Anatomy Ontology
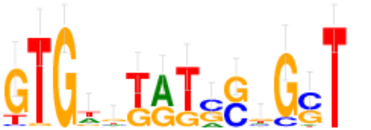	Score 26.1 Hits 21	anatomical structure development (1.2e-05)<br>system development (1.5e-05)<br>neuron migration (4.7e-05)<br>locomotion (5.6e-05)<br>cell migration (1.3e-04)	HSNR (8.2e-04)<br>HSN (8.2e-04)<br>HSNL (8.2e-04)<br>cholinergic neuron (8.9e-04)<br>somatic nervous system (9.8e-04)
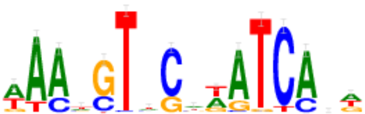	Score 26.1 Hits 284	growth (6.5e-19)<br>cytoplasm (1.2e-17)<br>small molecule metabolic process (3.8e-15)<br>locomotion (9.1e-15)<br>cytoplasmic part (2.0e-14)	ventral pharyngeal ganglion (post) (3.4e-04)<br>posterior lateral right ganglion (1.1e-03)<br>PVDR (1.7e-03)<br>PVD (1.7e-03)<br>PVDL (1.7e-03)
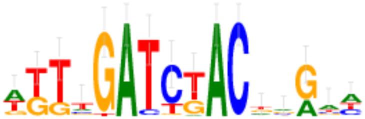	Score 26.1 Hits 22	ubiquitin-dependent protein catabol (4.0e-10)<br>modification-dependent macromolecul (4.0e-10)<br>modification-dependent protein cata (4.0e-10)<br>proteolysis involved in cellular pr (6.7e-10)<br>cellular protein catabolic process (7.1e-10)	
